# Cell-Surface Proteomics Identifies Differences in Signaling and Adhesion Protein Expression between Naive and Primed Human Pluripotent Stem Cells

**DOI:** 10.1016/j.stemcr.2020.03.017

**Published:** 2020-04-16

**Authors:** Katarzyna Wojdyla, Amanda J. Collier, Charlene Fabian, Paola S. Nisi, Laura Biggins, David Oxley, Peter J. Rugg-Gunn

**Affiliations:** 1 Epigenetics Programme, The Babraham Institute, Cambridge, UK; 2 Mass Spectrometry Facility, The Babraham Institute, Cambridge, UK; 3 Bioinformatics Group, The Babraham Institute, Cambridge, UK; 4 Wellcome-Medical Research Council Cambridge Stem Cell Institute, Cambridge, UK

**Keywords:** pluripotent stem cell, embryonic, reprogramming, cell state, JAK-STAT, cell-surface markers, proteomics, plasma membrane, human

## Abstract

Naive and primed human pluripotent stem cells (hPSC) provide valuable models to study cellular and molecular developmental processes. The lack of detailed information about cell-surface protein expression in these two pluripotent cell types prevents an understanding of how the cells communicate and interact with their microenvironments. Here, we used plasma membrane profiling to directly measure cell-surface protein expression in naive and primed hPSC. This unbiased approach quantified over 1,700 plasma membrane proteins, including those involved in cell adhesion, signaling, and cell interactions. Notably, multiple cytokine receptors upstream of JAK-STAT signaling were more abundant in naive hPSC. In addition, functional experiments showed that FOLR1 and SUSD2 proteins are highly expressed at the cell surface in naive hPSC but are not required to establish human naive pluripotency. This study provides a comprehensive stem cell proteomic resource that uncovers differences in signaling pathway activity and has identified new markers to define human pluripotent states.

## Highlights

• Direct measurement of plasma membrane proteins in two human pluripotent states
• Proteomic resource contains quantitative analysis of signaling and adhesion proteins
• Multiple cytokine receptors upstream of JAK-STAT pathway identified in naive cells
• Functional experiments reveal FOLR1 and SUSD2 are not required for naive pluripotency

## Graphical Abstract

**Figure:**
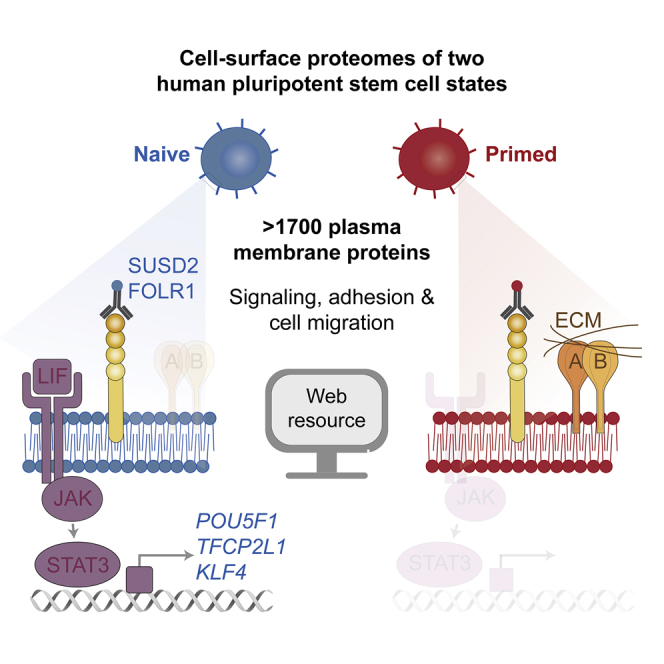

## Teaser

Wojdyla et al. use plasma membrane profiling to directly measure cell-surface protein expression in naive and primed human pluripotent stem cells. This comprehensive proteomic resource quantifies shared and state-specific abundance of signaling, adhesion, and cell migration proteins, thereby identifying new cell-surface markers and leading to a better understanding of how pluripotent cells communicate and interact with their microenvironments.

## Introduction

Recent reports have identified conditions that can stabilize human pluripotent stem cells (hPSC) in different states that hold distinct molecular and functional properties (Guo et al., 2017, Takashima et al., 2014, Theunissen et al., 2014). At the two ends of the pluripotency spectrum are naive hPSC that recapitulate the pre-implantation human epiblast, and conventionally grown primed hPSC that more closely align to the post-implantation epiblast (Dong et al., 2019). These cell types can provide informative models to study the earliest stages of human development and the control of pluripotent cell identity (Davidson et al., 2015, Weinberger et al., 2016).

Naive and primed hPSC differ substantially in their signaling responses, cell morphology, and growth requirements in culture, suggesting that they interact with and respond differently to their microenvironments (Collier and Rugg-Gunn, 2018). Although genomic and transcriptomic studies have provided a rich molecular characterization of naive hPSC, there is a lack of detailed information about cell-surface protein expression in these cells that could inform about this relationship. Cell-surface proteins are of particular interest due to their critical roles in cell communication and interactions (da Cunha et al., 2009), and their expression is typically more variable between cell types than other protein classes (Schwanhäusser et al., 2011). A quantitative measurement of the cell-surface proteome could provide information about how cells respond to external cues that control processes, such as growth and differentiation, which are critical to the function of pluripotent cell types. Profiling cell-surface proteins in naive hPSC could also identify markers that would help with their characterization and aid methods to accurately distinguish them from primed hPSC and other cell types that are required to resolve complex populations during cell state transitions (Collier and Rugg-Gunn, 2018, Goodwin et al., 2020, Trusler et al., 2018). In addition, strategies for enriching or eliminating target cell types within a population often rely on the known expression patterns of cell-surface markers and mapping surface proteomes is, therefore, a prerequisite for applying these approaches in naive and primed hPSC (Ben-David et al., 2013, Choo et al., 2008, Tang et al., 2011).

The correlation between transcript levels and protein abundance is particularly low in relation to cell-surface proteins (Boheler et al., 2014, Liu et al., 2016, Rugg-Gunn et al., 2012, Schwanhäusser et al., 2011), and also gene expression profiles do not provide information on post-translational modifications that can often provide epitopes for cell-type-specific antibodies (Ojima et al., 2015). Antibody-based screens in naive and primed hPSC have identified shared and state-specific cell-surface marker expression, although this approach is reliant on the availability of suitable antibodies (Collier et al., 2017). Accordingly, the direct measurement and quantitation of cell-surface proteins is desirable. Advances in mass spectrometry (MS)-based proteomic technologies have enabled the broad measurement of total proteomes of individual cell types (Aebersold and Mann, 2016), including of naive hPSC (Di Stefano et al., 2018). However, cell-surface proteins are typically underrepresented in these studies due to their low abundance and biochemical properties. In response to this limitation, several methods for measuring plasma membrane proteins by MS have been developed (Elschenbroich et al., 2010, Josic and Clifton, 2007) that are compatible with primed hPSC (Boheler et al., 2014, Dormeyer et al., 2008, Weldemariam et al., 2018).

In this study, we used plasma membrane profiling (PMP) (Weekes et al., 2012, Zeng et al., 2009) and tandem mass tags (TMT) (McAlister et al., 2012) to directly measure cell-surface protein expression in naive and primed hPSC. Our unbiased approach identified over 1,700 plasma membrane proteins, including those involved in cell adhesion, signaling, and cell interactions, thereby providing a comprehensive stem cell proteomic resource. As a first step toward exploring the potential role of identified cell-surface proteins, we generated knockout cell lines for two surface proteins that are expressed in naive hPSC, FOLR1, and SUSD2, and sought to examine any consequences on the acquisition of human naive pluripotency.

## Results

### Cell-Surface Proteomes of Human Pluripotent States

**Figure 1:**
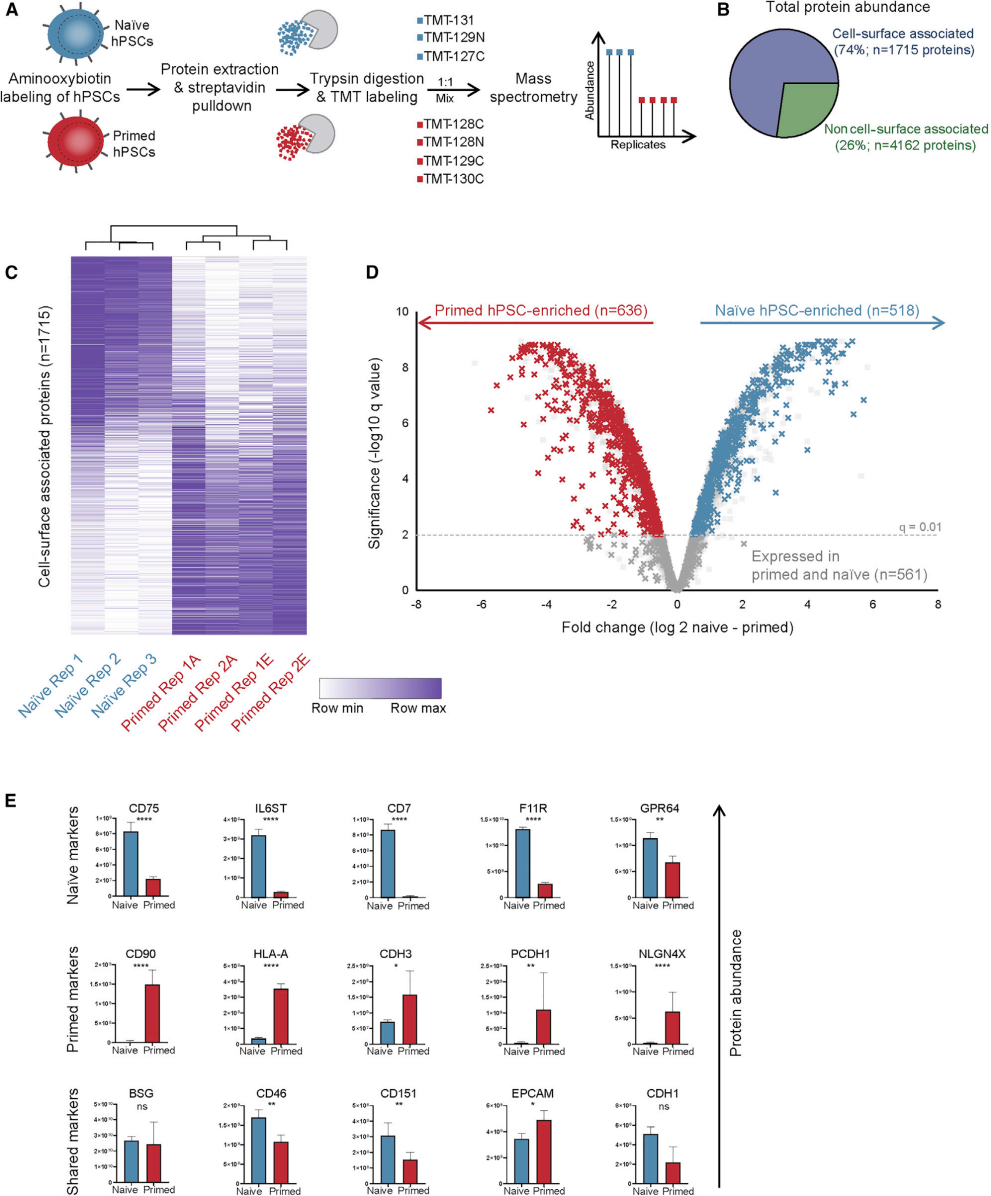
Cell-Surface Proteomics of Naive and Primed Human Pluripotent Stem Cells (A) Plasma membrane profiling and mass spectrometry to quantify cell-surface protein expression in hPSC. Naive H9 hPSC were maintained in t2iLGö on Matrigel-coated plates and primed H9 hPSC were maintained in TeSR-E8 on Vitronectin-coated plates. TMT, tandem mass tags. (B) Chart shows the proportion of total protein abundance for proteins annotated as plasma membrane/cell surface and proteins associated with all other subcellular locations (based on Uniprot KnowledgeBase). (C) Clustered heatmap shows the abundance of each measured cell-surface protein in all biological replicates. Values are normalized within each row. Primed hPSC samples are labeled according to their method of dissociation before PMP: A, Accutase; E, EDTA. (D) Volcano plot of fold change in cell-surface protein expression and the corresponding q value (LIMMA-moderated t test with Benjamini-Hochberg correction). Dashed line indicates the q = 0.01 cutoff that was applied to categorize differentially expressed proteins. (E) Protein abundance levels for several known state-specific or shared cell-surface markers. Data show the mean ± SD of three (naive) or four (primed) biological replicates and were compared using a LIMMA-moderated t test with Benjamini-Hochberg correction (ns, q > 0.05; ^∗^q < 0.05, ^∗∗^q < 0.01, ^∗∗∗^q < 0.001, ^∗∗∗∗^q < 0.0001). See also Figure S1 and Table S1.

We used plasma membrane protein enrichment and profiling to measure cell-surface protein expression in H9 naive and primed hPSC grown in feeder-free conditions (Figure 1A). Aminooxy-biotinylated plasma membrane proteins were prepared from multiple biological replicates and captured on streptavidin beads. Proteins were digested with trypsin and each sample was labeled with isobaric TMT to enable accurate relative quantitation. TMT-labeled samples were pooled, fractionated, and analyzed by MS. Pairwise comparisons of biological replicates revealed strong correlations between samples (average Pearson's correlation >0.98) (Figure S1A).

We attempted initially to harvest the cells using the non-proteolytic reagent EDTA, but naive hPSC were resistant to dissociation, and a brief treatment with the dissociation reagent Accutase was required to dislodge the cells. As harvesting the cells using the enzymes contained within Accutase might cleave cell-surface glycans and alter the abundance of cell-surface proteins, we first compared the proteomes obtained after releasing primed hPSC using either Accutase or EDTA (Figure S1B). The vast majority of proteins were quantitatively similar between the two conditions (r^2^ = 0.99), and fewer than 5% of proteins differed by more than 2-fold in expression (Figure S1C). Given this minor effect, we harvested naive hPSC with Accutase and combined samples to produce a high-quality dataset of three naive hPSC replicates and four primed hPSC replicates.

Applying cell-compartment annotations to the dataset revealed that ~75% of the total protein abundance corresponded to proteins with known or predicted plasma membrane or cell-surface localization, thereby confirming the efficient capture of cell-surface proteins (Figure 1B). A total of 1,715 plasma membrane-annotated proteins were quantified across naive and primed replicates. The remaining 25% of total protein abundance was derived from 4,162 proteins that are likely to interact with plasma membrane proteins or were isolated non-specifically. Altogether, this dataset provides a comprehensive stem cell proteomic resource (Table S1) that can be used to understand the cell interaction, communication, and signaling pathways controlling human pluripotent states. We have also created an online searchable tool for users to explore and visualize the proteomic data (http://www.bioinformatics.babraham.ac.uk/shiny/Wojdyla/ShinyProteomics/).

Relative quantitation of the 1,715 identified plasma membrane-annotated proteins showed that over two-thirds of the proteins were expressed in a cell-type-specific manner, demonstrating that naive and primed hPSC display distinct cell-surface proteomes (Figure 1C). Statistical analysis revealed that 518 cell-surface proteins were more abundant in naive compared with primed hPSC, and 636 were higher in primed (q < 0.01, LIMMA-moderated t test with multiple testing correction; Figure 1D). These sets of proteins contained several previously validated cell-surface markers. For example, naive-specific cell-surface proteins, such as CD75, CD130, CD7, F11R, and GPR64 were expressed at higher levels in naive compared with primed hPSC, and CD90, HLA-A, CDH3, PCDH1, and NLGN4X were more abundant in primed hPSC (Figure 1E) (Collier et al., 2017, Liu et al., 2017, O’Brien et al., 2017, Goodwin et al., 2020). Other proteins, such as BSG, CD46, CD151, EPCAM, and CDH1 that are expressed by naive and primed hPSC were measured at similar levels in both hPSC cell types (Figure 1E). As expected, we obtained a modest concordance when comparing mRNA levels and protein abundance (r^2^ = 0.54; Figure S1D), which is in line with previous studies that reported low correlations specifically for cell-surface proteins (Schwanhäusser et al., 2011). The correlation was also low when comparing our cell-surface proteome and a previous whole-cell proteome (r^2^ = 0.34; Figure S1E), which is due to the known underrepresentation and inaccurate quantitation of cell-surface proteins that afflicts whole-cell studies. Directly measuring plasma membrane protein abundance overcomes these limitations.

### Identification of Functional Classes

**Figure 2:**
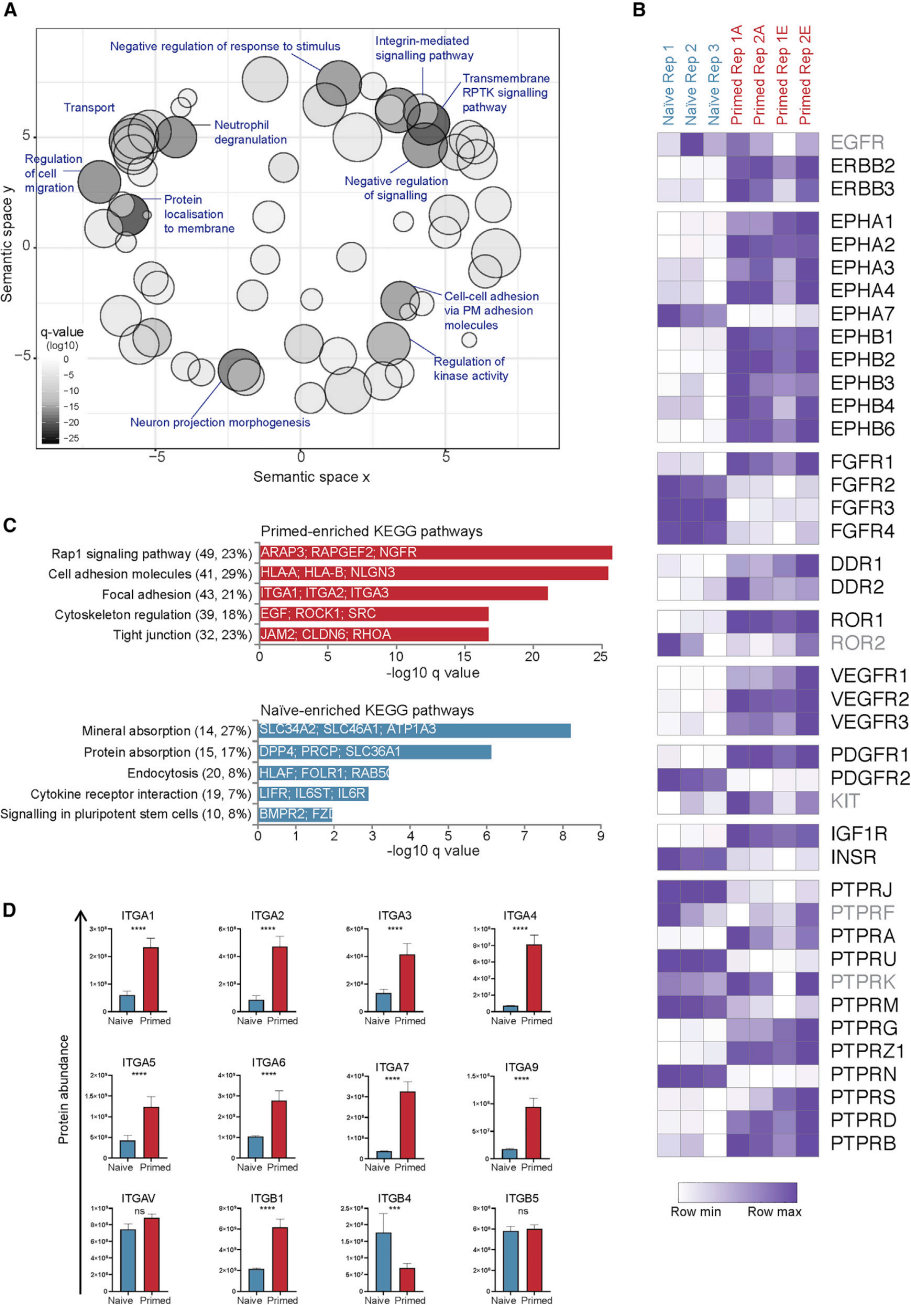
Functional Classes of State-Specific Cell-Surface Proteins (A) Scatterplot shows the GO terms that were overrepresented within the set of differentially expressed cell-surface proteins (n = 1,154). The two-dimensional space is derived by applying multidimensional scaling to a matrix of the GO terms' semantic similarities as calculated using REVIGO (Supek et al., 2011). Bubble size indicates the number of proteins that are associated with each term and bubble color indicates the q value of the enrichment (Fisher's exact test with Benjamini-Hochberg correction). See also Table S2. (B) Heatmap shows the abundance of cell-surface-localized receptor protein tyrosine kinases and receptor protein-tyrosine phosphatases. Values are normalized within each row. All proteins shown are differentially expressed between naive and primed hPSC except for the proteins in gray. Significance was tested using a LIMMA-moderated t test with Benjamini-Hochberg correction for multiple hypothesis testing; q < 0.05 was classified as significantly different. (C) Top-ranked KEGG pathways that are overrepresented within the set of primed-enriched proteins (upper) and naive-enriched proteins (lower) along with the q value of the enrichment for each pathway (Fisher's exact test with Benjamini-Hochberg correction). The values in brackets refer to the number of cell-surface proteins assigned to each pathway and the percentage coverage of each pathway. (D) Protein abundance levels for detected integrins. Data show the mean ± SD of three (naive) or four (primed) biological replicates and were compared using a LIMMA-moderated t test with Benjamini-Hochberg correction (ns, q > 0.05, ^∗∗∗^q < 0.001, ^∗∗∗∗^q < 0.0001). See also Figure S2.

We integrated Gene Ontology (GO) terms to examine the expression of proteins that belong to different functional classes (Figure 2A; Table S2). The transmembrane receptor protein tyrosine kinase (RPTK) signaling pathway was the most significantly enriched GO term within the signaling group (n = 91 proteins; q = 2 × 10^−27^, Fisher's exact test with multiple testing correction). Interestingly, most of these proteins were more abundant in primed than naive hPSC, including fibroblast growth factor receptor 1 (FGFR1), insulin-like growth factor 1 receptor (IGF1R), and most EPHRIN family members (Figure 2B). A small set of RPTK proteins showed the opposite trend, such as insulin receptor (INSR) and FGFR2, 3, and 4. In addition to RPTKs, receptor protein-tyrosine phosphatases were highly represented in the dataset with a more variable distribution between naive and primed states (Figure 2B).

We next analyzed the naive and primed hPSC datasets independently to identify cell-type-specific pathways (Figure 2C). In primed hPSC, RAP1 signaling was the most overrepresented pathway with 49 proteins assigned (q = 2 × 10^−26^). Notably, RAP1 activity is required for efficient self-renewal of primed hPSC (Li et al., 2010). Following that category, there were several GO terms related to cellular organization and communication, including cell adhesion molecules, focal adhesion, and tight junctions. Unexpectedly, our quantitative data showed that nearly all of the detected focal adhesion-associated integrins were more abundant in primed compared with naive hPSC (Figure 2D). Flow cytometry analysis of ITGA6 (CD49f) and ITGB1 (CD29) confirmed higher cell-surface expression in primed compared with naive hPSC (Figure S2A). Importantly, the expression of ITGA6 and ITGB1 was unaffected when primed hPSC were cultured on several different substrates (Figure S2B). Interestingly, ITGB4 was the only integrin in the proteomic dataset that was more abundant in naive than primed hPSC (Figure 2D). ITGB4 forms a dimer with ITGA6 to interact with laminin, and we note that laminin is one of the few defined substrates that can successfully maintain naive hPSC (Takashima et al., 2014).

We also examined the expression of cell-surface proteins that are upstream of transforming growth factor β (TGF-β) signaling, as this pathway is required for primed hPSC self-renewal (Vallier et al., 2004). Analysis of 18 plasma membrane proteins that are annotated within this pathway showed that, overall, their expression was moderately higher at the cell surface of primed compared with naive hPSC. We observed this trend for receptors of Activin/Nodal, such as ACVR2A and ACVR2B, and for TGF-β receptors, such as TGFBR1 (Table S1). Interestingly, expression of the BMP receptor BMPR2 was significantly higher in naive hPSC (by >3-fold; q = 8 × 10^−6^). Little is known about the effect of BMP on naive hPSC, although BMP signaling stabilizes naive pluripotency of mouse cells (Morikawa et al., 2016). The proteomic research presented here, therefore, provides valuable information about the differences in signaling and adhesion properties between human pluripotent states.

### JAK Signaling Is Required to Establish and Maintain Naive hPSC Proliferation and Gene Expression Program

In contrast to primed hPSC, only a small number of identified pathways were assigned to naive-enriched proteins. One of these pathways was cytokine-cytokine receptor interaction (n = 19; q = 1 × 10^−3^), which contained proteins, such as LIF receptor subunit alpha (LIFR), interleukin 6 signal transducer (IL6ST, CD130), and other receptors that activate JAK-STAT signaling (Figure 2C). GO analysis confirmed that the JAK-STAT3 signaling pathway was overrepresented among naive-enriched proteins (n = 11, q = 0.02; Table S2).

**Figure 3:**
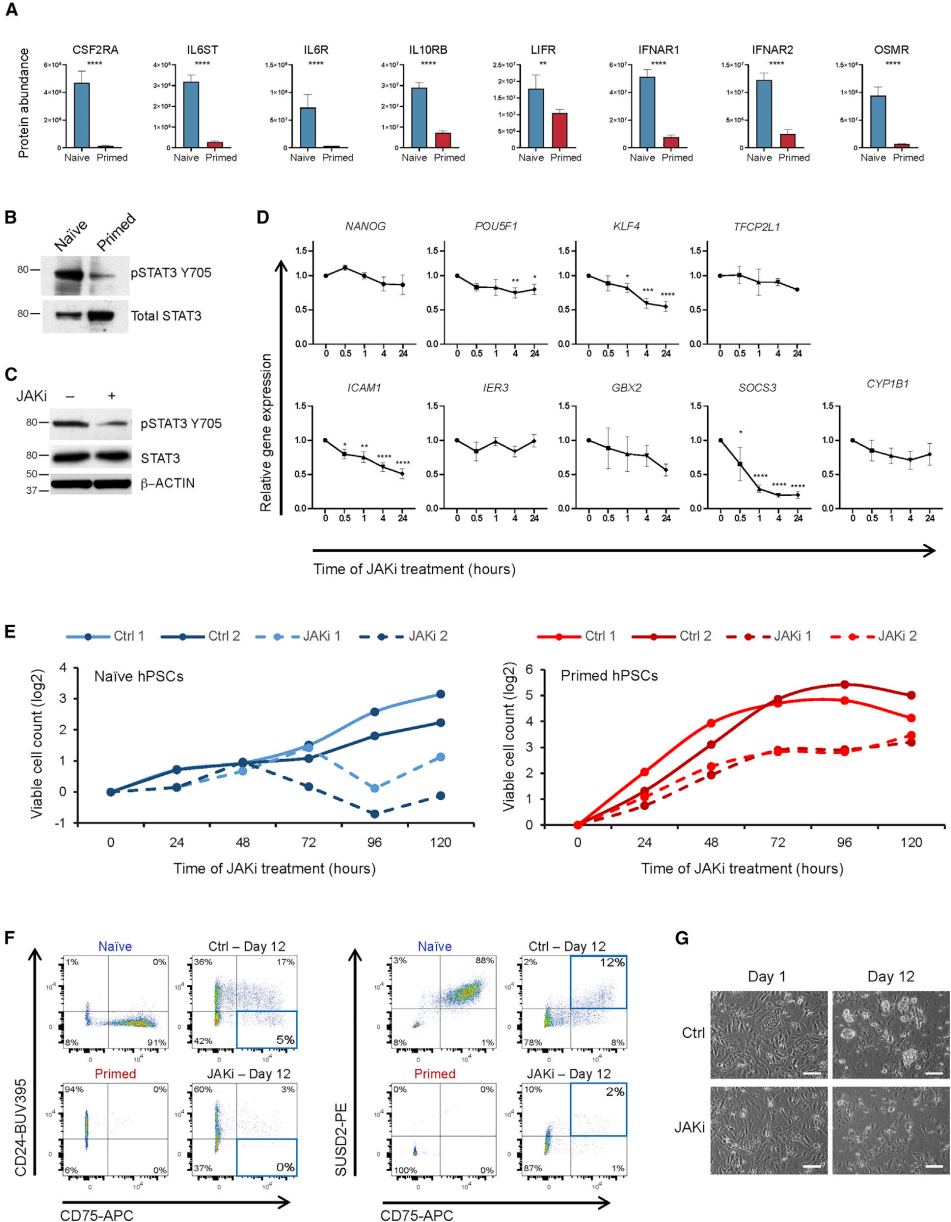
JAK-STAT3 Signaling Is Required for Primed to Naive Reprogramming and the Maintenance of Naive Pluripotency (A) Protein abundance levels for cytokine receptors that can activate JAK-STAT3 signaling. Data show the mean ± SD of three (naive) or four (primed) biological replicates and were compared using a LIMMA-moderated t test with Benjamini-Hochberg correction (^∗∗^q < 0.01, ^∗∗∗∗^q < 0.0001). (B) Western blot shows phosphorylated and total levels of STAT3 protein. Mass, kDa. Data in (B–E) were collected from naive H9 hPSC maintained in t2iLGö on Matrigel-coated plates and primed H9 hPSC maintained in TeSR-E8 on Vitronectin-coated plates. Results are representative of three biological replicates. (C) Western blot confirms the decrease in phosphorylated STAT3 after JAK inhibition in naive hPSC. Mass, kDa. (D) qRT-PCR analysis of gene expression levels in naive hPSC over a time course of JAK inhibition. Pluripotency-associated genes are shown in the top row and predicted JAK-STAT3 target genes are underneath (Chen et al., 2015). Data show the mean ± SD of three biological replicates and were compared with the 0 h time point using an ANOVA test with Dunnett's multiple comparisons test (ns, p > 0.05; ^∗^p < 0.05, ^∗∗^p < 0.01, ^∗∗∗^p < 0.001, ^∗∗∗∗^p < 0.0001). (E) Prolonged JAK inhibition (dashed lines) strongly impairs the proliferation of naive hPSC (left) with a modest effect on primed hPSC (right) compared with vehicle controls (solid line). Data are shown for two independent experiments. (F) Flow cytometry plots of live, human cells at day 12 of primed to naive hPSC reprogramming using Chemical Resetting conditions in the absence (Ctrl) and presence (JAKi) of JAK inhibitor. The JAK inhibitor was applied between days 5 and 10 at a final concentration of 1 μM. CD75 and SUSD2 are naive-specific markers and CD24 is a primed-specific marker. Quadrants containing reprogrammed naive cells are boxed in blue. Established naive and primed hPSC are shown as controls. (G) Phase contrast images of cells at days 1 and 12 during primed to naive hPSC reprogramming in the absence and presence of JAK inhibitor. Scale bar, 100 μm. Results in (F) and (G) are representative of three biological replicates.

Pathway members upstream of JAK-STAT signaling, including CSF2RA, IL6ST, IL6R, and OSMR, showed strong naive-specific expression at the cell surface, whereas the abundance of other proteins, such as LIFR were only marginally different between the two pluripotent cell types (Figure 3A). We verified that adding LIF to primed hPSC had no effect on IL6ST and IL6R cell-surface expression (Figure S2C). LIF-mediated activation of JAK-STAT3 signaling is critical for the establishment and maintenance of naive mouse pluripotent stem cells (Hall et al., 2009, van Oosten et al., 2012); however, this requirement has not been studied in naive hPSC cultured under stringent conditions. Consistent with the differential expression of upstream activators of the JAK-STAT3 pathway, western blot analysis revealed that the levels of active STAT3, marked by Y705 phosphorylation (pSTAT3), are substantially higher in naive compared with primed hPSC (Figure 3B). Applying a pan-JAK inhibitor to naive hPSC caused a decrease in pSTAT3 signal (Figure 3C).

We examined the effect of JAK inhibition and decreased pSTAT3 levels on the expression of predicted downstream genes in naive hPSC by time course qRT-PCR. *KLF4*, *ICAM1*, and *SOCS3* were significantly downregulated in the presence of JAK inhibition, *TFCP2L1* and *GBX2* were moderately reduced, and *IER3* and *CYP1B1* were unaffected (Figure 3D). Secondary effects were also observed on non-STAT3 target genes, including a decrease in *POU5F1* levels (Figure 3D). To determine whether the gene expression changes could be associated with an altered cell phenotype, we measured cell proliferation over 5 days of JAK inhibition. We found that JAK inhibition caused a strong reduction in the number of viable naive hPSC and a modest effect on primed hPSC (Figure 3E). Finally, we investigated whether JAK signaling is required to establish naive hPSC by inducing primed to naive hPSC reprogramming in the presence of a JAK inhibitor. Flow cytometry analysis revealed that cells exposed to a JAK inhibitor failed to reprogramme to the naive state (Figure 3F) and, using phase microscopy, we observed extensive cell death and few naive hPSC colonies in the JAK inhibitor-treated cultures (Figure 3G). Taken together, these results lead us to conclude that active JAK-STAT3 signaling is required for the establishment and maintenance of naive hPSC.

### An Expanded Set of Naive-Specific Cell-Surface Proteins

**Figure 4:**
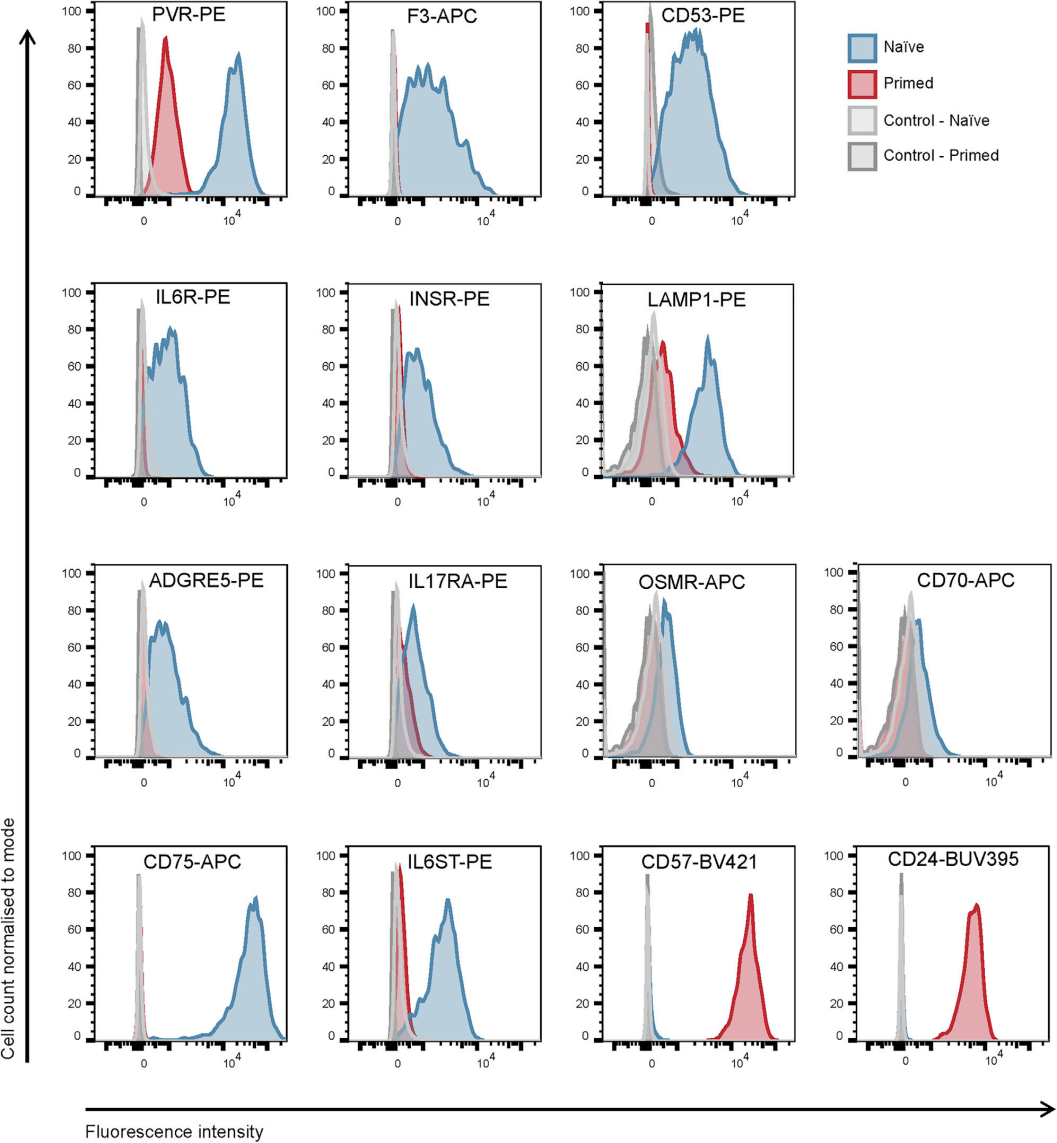
Antibody-Based Validations Confirm Naive-Specific Expression of Cell-Surface Proteins Histograms of flow cytometry analysis show separation between naive and primed H9 hPSC for several newly identified cell-surface proteins. As a positive control for the assay, CD75 and IL6ST (CD130), which are naive-specific cell-surface markers, and CD57 and CD24, which are primed-specific cell-surface markers were also examined (Collier et al., 2017). Naive H9 hPSC were maintained in t2iLGö on Matrigel-coated plates and primed H9 hPSC maintained in TeSR-E8 on Vitronectin-coated plates. Results are representative of at least three biological replicates. See also Figures S2–S4 and S6.

To discover new naive-specific markers, we used antibody-based assays to examine 22 cell-surface proteins that had >3-fold increase in protein abundance in naive compared with primed hPSC. Flow cytometry analysis of naive and primed hPSC confirmed clear, differential expression for 12 out of 22 proteins, with well-separated cell populations. Ten proteins were detected only at low levels or not detected above controls, potentially due to poor compatibility of the antibodies with flow cytometry or the absence of accessible epitopes. Antibody reactivity to PVR (CD155), F3 (CD142), and CD53 produced the best separation between naive and primed hPSC populations (Figure 4), similar to previously identified naive-specific markers, such as CD75 and IL6ST (CD130) (Collier et al., 2017). Additional, newly uncovered proteins, including IL6R (CD126), INSR (CD220), LAMP1 (CD107a), ADGRE5 (CD97), IL17RA (CD217), OSMR, and CD70 gave a reasonable separation in signal between cell types (Figure 4). We confirmed these results using additional hPSC lines, including the embryo-derived naive line HNES1 and the induced PSC primed line HDF (Figure S3). Importantly, the state-specific expression of each marker was preserved when hPSC were cultured on different substrates, including fibroblast cells, Matrigel, and Laminin (Figure S4). This validated set of proteins substantially increases the number of known markers that can discriminate between naive and primed hPSC.

To investigate whether the changes in expression of the identified markers recapitulate the developmental progression from pre-implantation to post-implantation epiblast, we examined several published transcriptional datasets, including hPSC capacitation (Rostovskaya et al., 2019), human epiblast cells (Xiang et al., 2020, Zhou et al., 2019), and primate epiblast cells (Nakamura et al., 2016). This analysis showed that transcript and protein levels correlated well for several of the naive-specific markers and, of those, genes such as *IL6R*, *SUSD2*, *CD53*, and *FOLR1*, are downregulated during pre-implantation to post-implantation epiblast development in human and primate embryos (Figure S5). These findings confirm that the identified naive-specific markers generally reflect developmental stage-specific differences *in vivo*.

### The Folate Receptor FOLR1 Is a New Cell-Surface Marker of Naive hPSC and Is Dispensable for Primed to Naive hPSC Reprogramming

**Figure 5:**
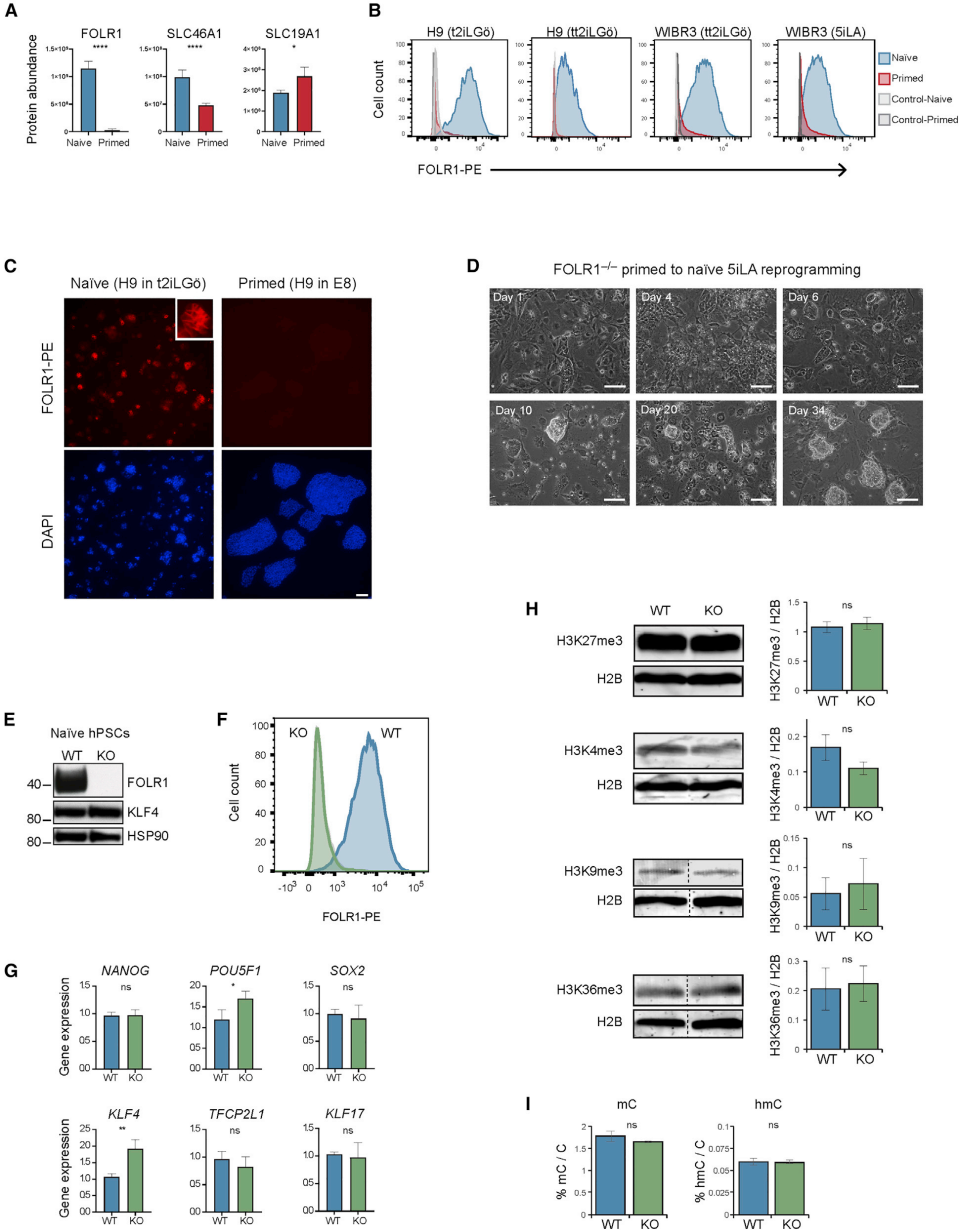
FOLR1 Is Highly Expressed in Naive hPSC but Is Not Required for Primed to Naive Reprogramming or Maintenance of the Naive State (A) Cell-surface abundance of detected folate receptors and transporters in naive and primed hPSC. Data show the mean ± SD of three (naive) or four (primed) biological replicates and were compared using a LIMMA-moderated t test with Benjamini-Hochberg correction (^∗^q < 0.05, ^∗∗∗∗^q < 0.0001). (B) Flow cytometry analysis confirms higher surface-localized FOLR1 expression in naive compared with primed hPSC. Left, H9 hPSC cultured in t2iLGö (naive) and E8 (primed); center left, H9 hPSC in tt2iLGö (naive) and E8 (primed); center right, WIBR3 hPSC in tt2iLGö (naive) and KnockOut serum replacement (KSR)-containing media (primed); right, WIBR3 in 5iLA (naive) and KSR-containing media (primed). Results are representative of three biological replicates. (C) Immunofluorescent microscopy images of FOLR1-PE and DAPI signals. Scale bar, 100 μm. Inset shows a magnified view of an individual naive colony. (D) Phase contrast images of FOLR1-deficient hPSC undergoing primed to naive 5iLA-mediated reprogramming. Scale bar, 100 μm. (E) Non-reducing western blot confirms the absence of FOLR1 protein in FOLR1-deficient naive hPSC. Mass, kDa. Results are representative of three biological replicates. (F) Flow cytometry analysis confirms the absence of FOLR1 signal in FOLR1-deficient (knockout [KO]) naive hPSC compared with parental (wild-type [WT]) naive hPSC. (G) qRT-PCR analysis of gene expression levels in parental (WT) naive hPSC and FOLR1-deficient (KO) naive hPSC. Data show the mean ± SD of three biological replicates and were compared using an unpaired, two-sided t test (ns, q > 0.05; ^∗^q < 0.05, ^∗∗^q < 0.01). (H) Western blots show the global levels of histone modifications in parental (WT) naive hPSC and FOLR1-deficient (KO) naive hPSC. H2B serves as a loading control. Charts show the quantitation of western blot fluorescence signals. Data show the mean ± SD of three technical replicates. (I) Quantitation by mass spectrometry of global mC and hmC levels in parental (WT) and FOLR1-deficient (KO) naive hPSC. Data show the mean ± SD of three technical replicates and were compared using an unpaired, two-sided t test. See also Figures S6A and S6B.

FOLR1 was ranked number eight on the list of naive-enriched proteins, with a 29-fold higher cell-surface expression in naive compared with primed hPSC (Table S1). FOLR1 is a high-affinity folate receptor and, together with FOLR2 and FOLR3, binds and internalizes folate, thereby providing methyl groups for one-carbon metabolism and methylation of DNA and histones (Lintas, 2019). Folate deficiency during embryonic development is associated with neural tube defects, but there is no information about its role at the early blastocyst stage and its impact on pluripotency. FOLR1 was the only folate receptor identified within our cell-surface proteomics dataset (Figure 5A), and we detected the expression of two folate transporters SLC46A1 and SLC19A1 (Figure 5A). We used an antibody to confirm naive-specific cell-surface expression of FOLR1 in multiple cell lines and growth conditions using flow cytometry (Figures 5B and S6A) and immunofluorescence microscopy (Figure 5C).

To investigate whether FOLR1 has a functional role in naive hPSC, we used CRISPR-Cas9 to generate FOLR1-deficient primed hPSC and attempted to reprogramme the mutant cells to a naive state. After 10 days of reprogramming, colonies with clear naive hPSC colony morphology were observed in the FOLR1-deficient samples (Figure 5D). By day 34 of reprogramming, corresponding to five passages, stable naive hPSC cultures were apparent with dome-shaped morphology (Figure 5D). Western blot experiments and flow cytometry analysis confirmed the absence of FOLR1 signal in FOLR1-deficient naive hPSC (Figures 5E and 5F). Transcriptional analysis showed that *FOLR3* expression was significantly higher in FOLR1-deficient naive hPSC compared with parental controls, raising the possibility that FOLR3 might partially compensate for the loss of FOLR1, although this is unlikely as *FOLR3* transcript levels remained low overall (Figure S6B). *FOLR2* levels were not significantly different in the absence of FOLR1 (Figure S6B). Taken together, these results suggest that FOLR1 is not required for primed to naive hPSC reprogramming.

To characterize the FOLR1-deficient naive hPSC in more detail, we next asked whether gene expression levels are altered in the mutant cells. Using qRT-PCR, we observed no difference in the expression of *NANOG*, SOX2, *TFCP2L1*, or *KLF17* between FOLR1-deficient naive hPSC and parental controls (Figure 5G). *POU5F1* and *KLF4* levels were slightly increased in the knockout cells, although KLF4 protein levels were unchanged (Figures 5E and 5G).

Because the receptor-mediated internalization of folate provides a methyl donor source for protein and DNA methylation, we hypothesized that the deletion of FOLR1 could reduce methylation levels of key epigenetic modifications. We used western blotting to measure the global abundance of several prominent histone methylation marks. We found there were no significant differences in H3K27me3, H3K4me3, H3K9me3, or H3K36me3 levels between FOLR1-deficient naive hPSC compared with parental controls (Figure 5H). Similarly, mass spectrometric quantitation revealed there was no significant difference in either mC or hmC levels in the FOLR1-deficient cells compared with controls (Figure 5I). Altogether, these findings reveal that FOLR1 is not required for primed to naive hPSC reprogramming or for the maintenance of naive pluripotency.

### SUSD2 Is Highly Expressed in Naive hPSC but Is Not Required to Establish Naive Pluripotency

**Figure 6:**
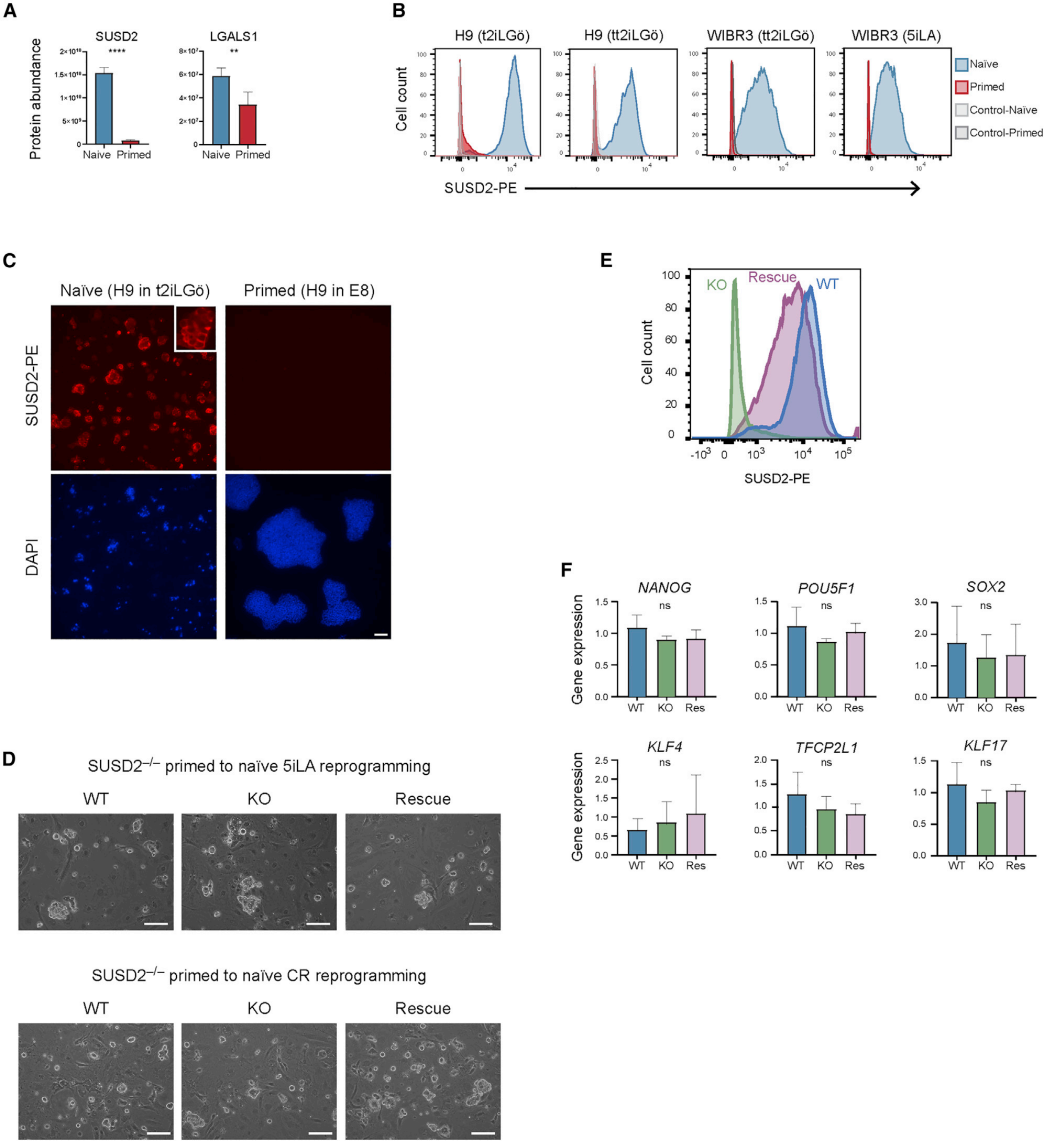
SUSD2 Is Highly Expressed in Naive hPSC but Is not Required for Reprogramming to Naive Pluripotent State (A) Cell-surface abundance of SUSD2 and LGALS1. Data show the mean ± SD of three (naive) or four (primed) biological replicates and were compared using a LIMMA-moderated t test with Benjamini-Hochberg correction (^∗^q < 0.05, ^∗∗∗∗^q < 0.0001). (B) Flow cytometry analysis confirms higher surface-localized SUSD2 expression in naive compared with primed hPSC. Left, H9 hPSC cultured in t2iLGö (naive) and E8 (primed); center left, H9 hPSC in tt2iLGö (naive) and E8 (primed); center right, WIBR3 hPSC in tt2iLGö (naive) and KSR-containing media (primed); right, WIBR3 in 5iLA (naive) and KSR-containing media (primed). Results are representative of three biological replicates. (C) Immunofluorescent microscopy images of SUSD2-PE and DAPI signals. Scale bar, 100 μm. Inset shows a magnified view of an individual naive colony. (D) Phase contrast images of SUSD2-deficient naive hPSC after reprogramming in 5iLA (upper) or Chemical Resetting (lower) conditions. Scale bar, 100 μm. (E) Flow cytometry analysis confirms the absence of SUSD2 signal in SUSD2-deficient (KO) naive hPSC compared with parental (WT) and KO + SUSD2 expression (Rescue) naive hPSC. (F) qRT-PCR analysis of gene expression levels in parental (WT), SUSD2-deficient (KO), and rescue (Res) naive hPSC. Data show the mean ± SD of three biological replicates and were compared using an unpaired, two-sided t test (ns, q > 0.05; ^∗^q < 0.05, ^∗∗^q < 0.01). Data in (D–F) are from one KO and one rescue hPSC line. Similar results were obtained from an alternative pair of KO and rescue lines (data not shown). See also Figures S6C and S6D.

SUSD2 is a plasma membrane protein thought to be a tumor suppressor acting via G1 cell-cycle arrest, but its role in early human development is unknown. SUSD2 was number 30 on the list of naive-enriched proteins with an 18-fold higher cell-surface expression in naive compared with primed hPSC (Figure 6A). The SUSD2 interaction partner LGALS1 was also more abundant in naive cells (Figure 6A). We confirmed the naive-specific cell-surface expression of SUSD2 by flow cytometry and immunofluorescence microscopy in two different hPSC lines and multiple culture conditions (Figures 6B, 6C, and S6C). A recent report also identified SUSD2 as a specific marker of naive hPSC and pre-implantation epiblast cells (Bredenkamp et al., 2019a).

As SUSD2 is highly expressed in naive hPSC, we set out to investigate whether SUSD2 is required for primed to naive hPSC reprogramming. We used CRISPR-Cas9 to generate SUSD2-deficient primed hPSC. As an additional control, we introduced an *SUSD2* expression plasmid into the mutant cell lines and confirmed that the SUSD2 signal at the cell surface was similar to the levels detected in wild-type naive hPSC (Figure S6D). We initiated primed to naive hPSC reprogramming using 5iLA conditions, and after several days multiple colonies appeared with characteristic domed-shaped morphology in the SUSD2-deficient and control cell lines (Figure 6D, upper). We tested if there could be differences in SUSD2 necessity when using an alternative reprogramming protocol, however SUSD2-deficient cells were successfully reprogrammed using Chemical Resetting conditions (Figure 6D, lower). Flow cytometry analysis confirmed the absence of the SUSD2 signal in SUSD2-deficient naive hPSC (Figure 6E). Finally, we observed similar expression levels of naive-specific marker genes, such as *KLF4*, *KLF17*, and *TFCP2L1*, in SUSD2-deficient naive hPSC and control cell lines (Figure 6F). Taken together, these results confirm that SUSD2 is a specific marker of naive hPSC, but is not required to establish naive pluripotency.

## Discussion

Our comparative proteomic analysis showed for the first time that the abundance of plasma membrane proteins was quantitatively distinct between naive and primed hPSC. Applying plasma membrane enrichment and TMT labeling enabled the direct measurement of protein abundance at the cell surface, which overcomes some of the limitations that are associated with inferring protein expression from transcriptional data, antibody screens, and whole-cell proteomics. This applies particularly when comparing cell types where transcript or whole-cell protein levels are often not predictive of differences at the plasma membrane due to post-translational processing. The final dataset contains quantitative values on over 1,700 proteins that are expressed at the cell surface, providing major advances in identifying new cell-type-specific markers and uncovering novel target proteins with potential functional roles in developmental and stem cell biology.

Our study provides direct validation for several cell-surface proteins that are currently used to distinguish between naive and primed hPSC (Collier et al., 2017, Liu et al., 2017, O’Brien et al., 2017), and found additional markers that are expressed by naive hPSC, such as F3, PVR, and CD53. Tracking the expression of these proteins using antibodies will provide a useful readout of naive hPSC differentiation and reprogramming. For example, the downregulation of CD53 expression was recently used to examine naive hPSC differentiation during lineage specification (Linneberg-Agerholm et al., 2019). Notably, several of the top-ranked naive-enriched proteins are poorly characterized, including FAM151A and FAM174C, and their identification provides interesting new avenues for pluripotent stem cell research. For example, *FAM1*74C is a recently evolved gene present only in higher primates that encodes a secreted protein of unknown function (Wang et al., 2006), and so naive hPSC should provide a useful cell model in which to study this uncharacterized protein. In addition, we found that the surface protein CLAUDIN-6 is differentially expressed with a higher abundance in primed compared with naive hPSC. This opens up the possibility of applying a published strategy that eliminates CLAUDIN-6-expressing cells to selectively remove primed hPSC from mixed pluripotent cell cultures (Ben-David et al., 2013). This approach could be used effectively during cell reprogramming to obtain purified populations with a higher proportion of naive hPSC.

Our stem cell proteomic resource contains detailed information about the expression of cell-surface ligands and receptors that is important for understanding the regulation of cell behavior in developmental processes. One major category of proteins that we measured was integrins, which mediate cell-matrix and cell-cell adhesion interactions (Harburger and Calderwood, 2009, Hynes, 2002). There are 24 integrins in humans, which arise from the heterodimeric association between one of 18 α subunits and 8 β subunits. Different combinations of the α and β polypeptides form complexes that vary in their ligand-binding specificities. We detected nine α subunits and three β subunits in our study. The three β subunits had interesting expression patterns whereby β1 expression was substantially higher in primed hPSC, β4 showed the opposite pattern, and β5 was expressed equally in both cell types. β1 interacts with many α subunits and typically binds to collagens, laminins, and fibronectin. β4 binds predominantly with α6 and this heterodimer interacts with laminins (Chen et al., 2008, Lee, 1992) and activates several signaling molecules, including NRG1, IGF1, IGF2, SHC1, and IRS2 (Cedano Prieto et al., 2017, Fujita et al., 2012). In contrast, other β integrins, including β1 and β5, activate alternative pathways, such as FAK and ILK (Grashoff et al., 2004, Guo and Giancotti, 2004). Through these processes, integrins provide information to the cells about their location, local environment, adhesive state, and surrounding matrix, and transduce signals that regulate gene expression and cell growth (Gahmberg et al., 2009). It is possible that the cell-surface abundance of integrins could be influenced by the different substrates used for maintaining naive and primed hPSC; however, we examined this possibility for two integrin proteins and found that their expression was unaffected when hPSC were cultured on different substrates. An important next step will be to test the function of integrin proteins in naive hPSC using blocking antibodies and small-molecule antagonists (Byron et al., 2009). Future studies could also use the new information on integrins and other surface-localized ligand expression within our dataset to design chemically defined substrates that are capable of supporting naive hPSC cultures (Lambshead et al., 2018).

GO analysis of the cell-type-specific protein expression identified several clear and unexpected functional classes. For example, cell-surface proteins expressed in primed hPSC were associated with RAP1 signaling. RAP1 is a small GTPase that is required in primed hPSC for efficient self-renewal and survival by controlling E-CADHERIN-mediated cell adhesion (Li et al., 2010). The loss of cell contacts causes the disruption of RAP1-E-CADHERIN signaling (Li et al., 2010), and this could partly underlie the sensitivity of primed hPSC to single-cell dissociation (Watanabe et al., 2007). RAP1 also modulates a range of signaling pathways in many cell types (Jaśkiewicz et al., 2018). It is interesting, therefore, that naive hPSC express lower levels of proteins that are associated with RAP1 signaling, suggesting this pathway is not required to sustain naive hPSC. Notably, naive hPSC are robust to single-cell dissociation and it will be important to investigate whether this is partly due to the difference in RAP1 and the regulation of E-CADHERIN. Conversely, the functional classes enriched within naive-specific cell-surface proteins included mineral absorption, solute carriers, and cytokine receptor interactions. In particular, plasma membrane-associated components of the LIF-JAK-STAT3 signaling pathway were more abundant in naive than primed hPSC, and STAT3 showed higher activation levels. Previous studies using non-stringent culture conditions have shown that hPSC acquire LIF dependency after treatment with media containing 3i (MEK, GSK3β, and BMP4 inhibitors) and LIF (Chan et al., 2013), or after enforced STAT3 activity in the presence of LIF and 2i (MEK and GSK3β inhibitors) (Chen et al., 2015). Here, we used stringent culture conditions to show that JAK signaling is required for the conversion of primed to naive hPSC, and also for the stable maintenance and proliferation of established naive hPSC. Defining the downstream effectors of JAK-STAT3 signaling in naive hPSC is an important line of research and the set of target factors could be compared with emerging data in other species to identify shared and species-specific modes of regulation (Bernardo et al., 2018, Bourillot et al., 2020, Ramos-Ibeas et al., 2019). Excitingly, we also detected in our proteomics experiment the expression of multiple cytokine receptors and ligands at the surface of naive hPSC that are currently understudied in human pluripotency, such as OSMR, CSF2RA, and IL10RB. Investigating the functional roles of these cell-surface proteins in activating JAK-STAT signaling in human pluripotent cells is a key future direction and one that could generate more stable conditions to maintain naive pluripotency.

FOLR1 and SUSD2 were highly ranked, naive-specific cell-surface proteins that were identified in our proteomic dataset. We showed that both proteins were expressed in naive hPSC cultured under different growth conditions, demonstrating that FOLR1 and SUSD2 can robustly and unambiguously discriminate between naive and primed hPSC. Tracking their expression using antibodies will provide new ways to monitor and study cell reprogramming events. A similar observation on SUSD2 expression was reported recently (Bredenkamp et al., 2019a) and the application of this marker to reprogramming (Bredenkamp et al., 2019b). We extended our analysis by demonstrating that neither FOLR1 nor SUSD2 were required to establish naive pluripotency, as knockout primed hPSC could be reprogrammed into a naive state. Folate endocytosis via FOLR1 provides one of the main donors of the methyl group required for the synthesis of S-adenosylmethionine, which in turn is needed for DNA and histone methylation. We hypothesized that DNA and histone methylation levels could be reduced by the absence of FOLR1 in naive hPSC, however, we detected unchanged levels of DNA methylation and several histone modifications. It is possible that FOLR1 deficiency could be compensated for by one of the alternative folate transporters. Nevertheless, our discovery of FOLR1 expression in naive hPSC opens up additional exciting research directions, for example, by examining whether other folate-dependent pathways, such as one-carbon metabolism, show differences between naive and primed hPSC.

Our study provides an invaluable resource for stem cell researchers by adding the cell-surface proteome as a new layer to the molecular characterization of pluripotency, highlighting the similarities and differences between two human pluripotent states. The proteomic dataset could be taken forward in several ways. Further analysis of the identified proteins at a functional level will uncover new pathways that control hPSC regulation. In addition, integrating phosphoproteomic information to connect the presence of receptors at the cell surface with their downstream signaling responses would provide a more complete picture of regulatory pathways in human pluripotent states.

## Experimental Procedures

### Cell Culture

H9, HNES1, and WIBR3 naive hPSC were maintained in t2iLGö, PGXL, and 5iLA media (Takashima et al., 2014, Theunissen et al., 2014, Guo et al., 2017). All naive hPSC were passaged by dissociation with Accutase every 3–4 days. H9, WIBR3, and HDF primed hPSC were maintained in TeSR-E8 or in KnockOut serum replacement-containing media. Primed cells were passaged using 0.5 mM EDTA or 200 U/mL Collagenase type IV. All hPSC were cultured in 5% O_2_, 5% CO_2_ at 37°C. See Supplemental Experimental Procedures for full details.

## Author Contributions

Conceptualisation, K.W., D.O. and P.J.R.-G.; Methodology, K.W.; Validation, K.W.; Formal analysis, K.W. and P.J.R.-G.; Investigation, K.W., A.J.C, C.F., P.S.N., L.B., D.O. and P.J.R.-G.; Data Curation, K.W.; Writing - Original Draft, K.W. and P.J.R.-G.; Writing - Review and Editing, K.W., A.J.C., C.F., P.S.N., L.B., D.O. and P.J.R.-G.; Visualization, K.W. and P.J.R.-G.; Supervision, D.O. and P.J.R.-G.; Project Administration, K.W. and P.J.R.-G.; Funding Acquisition, D.O. and P.J.R.-G.

## Accession Numbers

Raw data and the combined Proteome Discoverer search file have been deposited to the ProteomeXchange Consortium (http://proteomecentral.proteomexchange.org) via the PRIDE partner repository (Vizcaíno et al., 2013) with identifier PXD015359. The data are also available online at http://www.bioinformatics.babraham.ac.uk/shiny/Wojdyla/ShinyProteomics.

## Supplemental Information

Document S1. Supplemental Experimental Procedures and Figures S1–S6

Table S1. Full Dataset of Cell-Surface Proteomes in Naive and Primed hPSC, Related to Figure 1

Table S2. List of GO Terms that Are Associated with the Detected Cell-Surface Proteins, Related to Figure 2

Table S3. List of Antibodies Used, Related to Figure 4 and Supplemental Experimental Procedures

Document S2. Article plus Supplemental Information
