# Supplementary material for: Cell-Surface Proteomics Identifies Differences in Signaling and Adhesion Protein Expression between Naive and Primed Human Pluripotent Stem Cells
Source: Stem Cell Reports. 2020 Apr 16;14(5):972–88. doi: 10.1016/j.stemcr.2020.03.017 (PMC7220956; doi:10.1016/j.stemcr.2020.03.017)
Supplement: Document S1. Supplemental Experimental Procedures and Figures S1–S6 [file mmc1.pdf]

Stem Cell Reports, Volume *14*

Supplemental Information

Cell-Surface Proteomics Identifies Differences in Signaling and Adhesion Protein Expression between Naive and Primed Human Pluripotent Stem Cells

Katarzyna Wojdyla, Amanda J. Collier, Charlene Fabian, Paola S. Nisi, Laura Biggins, David Oxley, and Peter J. Rugg-Gunn

SUPPLEMENTAL FIGURE LEGENDS

Figure S1. QC of biological replicates and comparison to transcriptional and whole cell proteomic data sets, Related to Figure 1.

(A) Scatter plots show pairwise comparisons between biological replicates with Pearson correlation r^2 values. N1 refers to Naive Replicate 1 etc; P1A refers to Primed Replicate 1 harvested with Accutase; P1E refers to Primed Replicate 1 harvested with EDTA.

(B) Experimental design used to test the effect of colony dissociation methods on the measurement of cell-surface proteins.

(C) Scatter plot compares the mean abundance of cell-surface proteins in samples that were dissociated using Accutase or EDTA, with Pearson correlation r^2 value. Proteins with a difference in abundance >2-fold are marked in grey (n=76; 4.4%) and all other proteins are marked in black (n=1639; 95.6%).

(D) and (E) Scatter plots compare the fold difference in the abundance of cell-surface proteins between naive and primed hPSC measured in our PMP experiment with the corresponding values for (D) transcript (n=1413; data from (Takashima et al., 2014)) or (E) whole cell proteome (n=1078; data from (Di Stefano et al., 2018)) values. Pearson correlation r^2 values are shown. Naive hPSC were cultured in t2iLGö for all datasets.

Figure S1

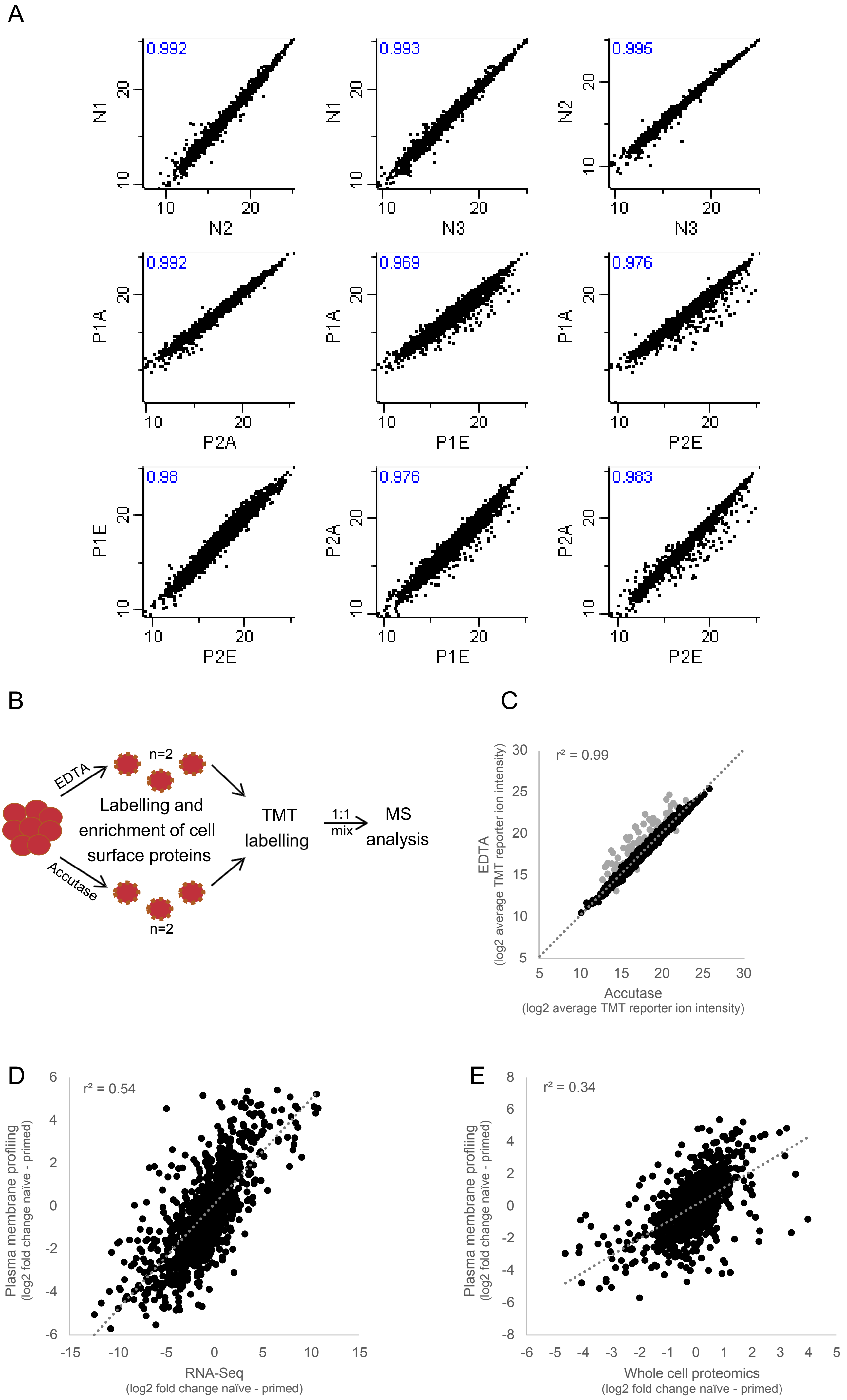
A
0.992
0.993
0.995
N1
N2
N3
0.992
0.969
0.976
P1A
P2A
P1E
P2E
0.98
0.976
0.983
B
EDTA
n=2
Labelling and enrichment of cell surface proteins
Accutase
TMT labelling
1:1 mix
MS analysis
C
r² = 0.99
EDTA
(log2 average TMT reporter ion intensity)
Accutase
(log2 average TMT reporter ion intensity)
D
r² = 0.54
Plasma membrane profiling
(log2 fold change naïve - primed)
RNA-Seq
(log2 fold change naïve - primed)
E
r² = 0.34
Plasma membrane profiling
(log2 fold change naïve - primed)
Whole cell proteomics
(log2 fold change naïve - primed)

Figure S2. Minimal effect of different substrates on ITGB1 and ITGA6 expression, and on LIF-receptor expression after the addition of LIF to primed hPSC, Related to Figures 2 and 4.

(A) Flow cytometry histograms of naive (t2iLGö) and primed (E8 media) H9 hPSC cultured on Laminin (left) or Matrigel (right) for CD29 (ITGB1) and CD49F (ITGA6).

(B) Flow cytometry histograms of primed H9 (left) and HDF (right) hPSC maintained in E8 media on either Vitronectin, Matrigel or Laminin for CD29 (ITGB1) and CD49F (ITGA6).

(C) Flow cytometry histograms of primed hPSC after seven days with and without LIF in the E8 media. IL6R and IL6ST expression remains low in both sets of primed hPSC samples, compared to naive hPSC. CD75 and CD24 provide validation of the naive and primed hPSC.

Results in this figure are representative of at least two biological replicates.

Figure S2

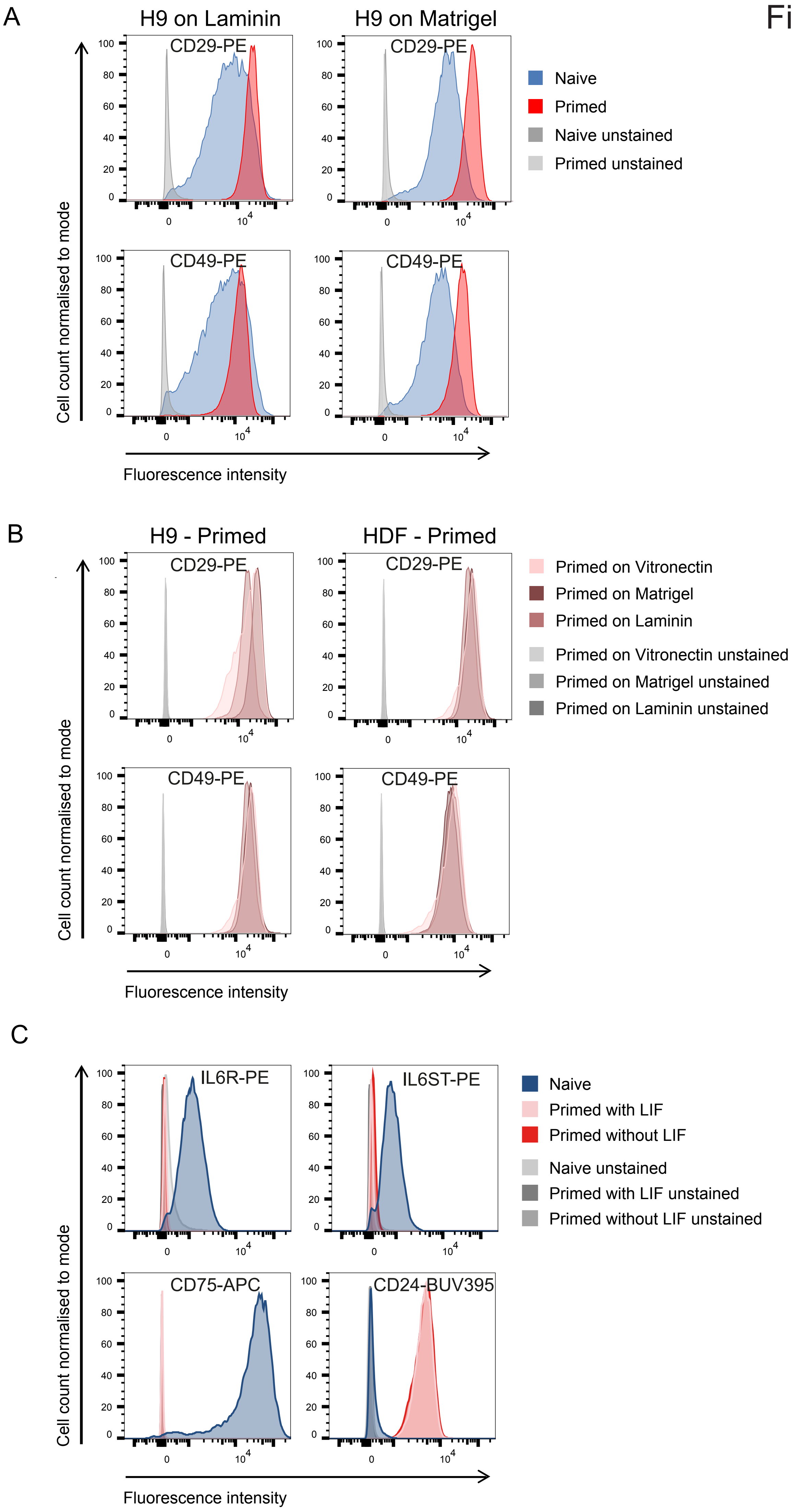

A
H9 on Laminin
H9 on Matrigel
CD29-PE
CD49-PE
Naive
Primed
Naive unstained
Primed unstained
Cell count normalised to mode
Fluorescence intensity
B
H9 - Primed
HDF - Primed
CD29-PE
CD49-PE
Primed on Vitronectin
Primed on Matrigel
Primed on Laminin
Primed on Vitronectin unstained
Primed on Matrigel unstained
Primed on Laminin unstained
Cell count normalised to mode
Fluorescence intensity
C
IL6R-PE
IL6ST-PE
CD75-APC
CD24-BUV395
Naive
Primed with LIF
Primed without LIF
Naive unstained
Primed with LIF unstained
Primed without LIF unstained
Cell count normalised to mode
Fluorescence intensity

Figure S3. Antibody-based validations confirm naïve-specific expression of cell-surface proteins in additional hPSC lines, Related to Figure 4.

Histograms of flow cytometry analysis using fluorophore-conjugated antibodies show separation in the fluorescence signal between naive (HNES1 line) and primed (HDF line) hPSC for several newly identified cell-surface proteins. As a positive control for the assay, CD75 and CD130, which are naive-specific cell-surface markers, and CD57 and CD24, which are primed-specific cell-surface markers were also examined (Collier et al., 2017). Naive HNES1 hPSC were maintained in PDXL media on Geltrex and primed HDF hPSC were maintained in TeSR-E8 on Vitronectin. Results in this figure are representative of at least two biological replicates.

Figure S3

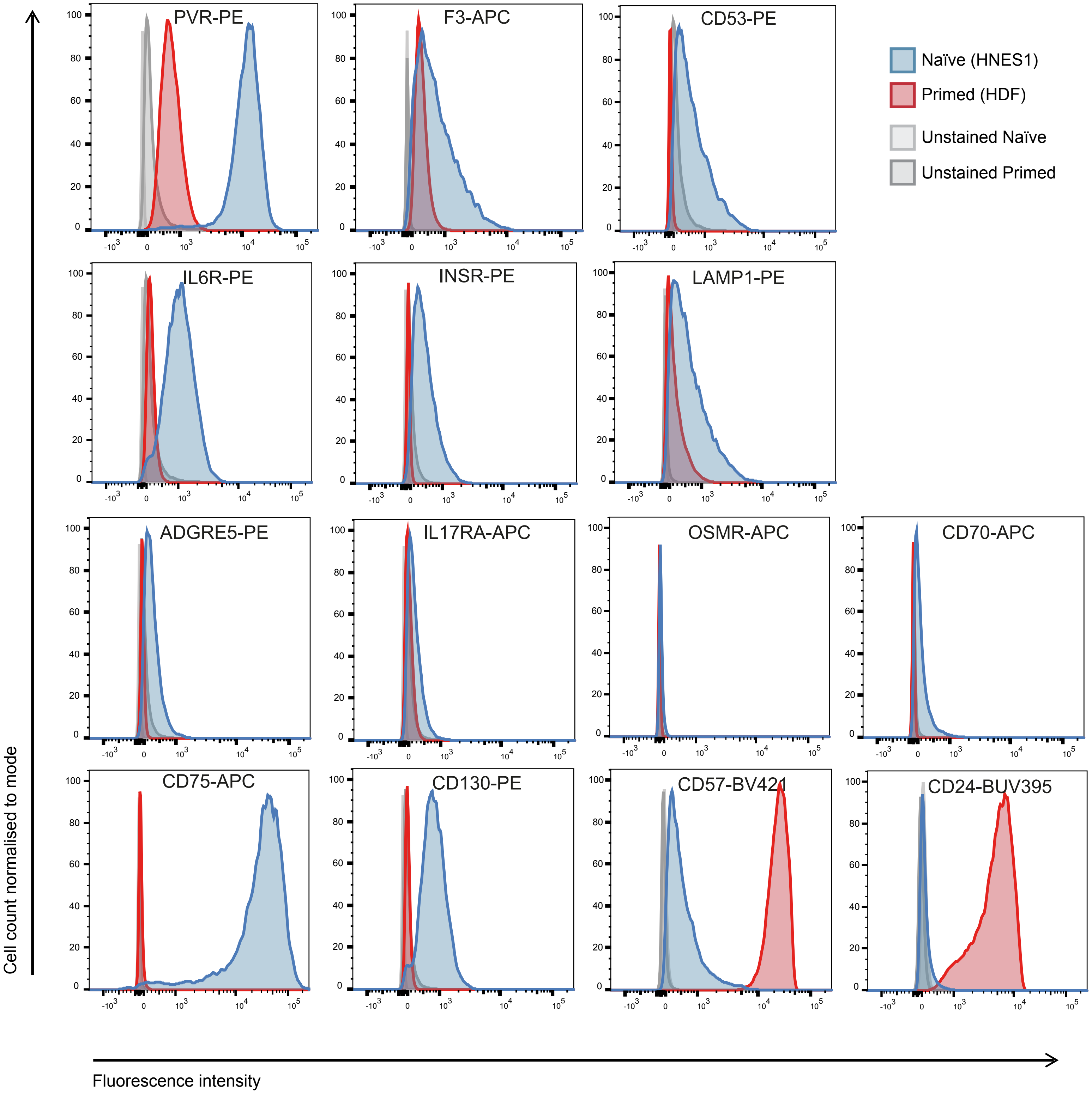

PVR-PE
F3-APC
CD53-PE
IL6R-PE
INSR-PE
LAMP1-PE
ADGRE5-PE
IL17RA-APC
OSMR-APC
CD70-APC
CD75-APC
CD130-PE
CD57-BV421
CD24-BUV395
Naïve (HNES1)
Primed (HDF)
Unstained Naïve
Unstained Primed
Cell count normalised to mode
Fluorescence intensity

Figure S4. Naïve-specific expression of cell-surface proteins is independent of substrate, Related to Figure 4.

Flow cytometry experiments show that the separation in the fluorescence signal of several identified cell-surface markers is independent of the substrate used to maintain the H9 naive and primed hPSC. Shown here are the results obtained when mitotically-inactivated embryonic fibroblasts (MEFs), Matrigel and Laminin were used as substrates. Results in this figure are representative of at least two biological replicates.

Figure S4

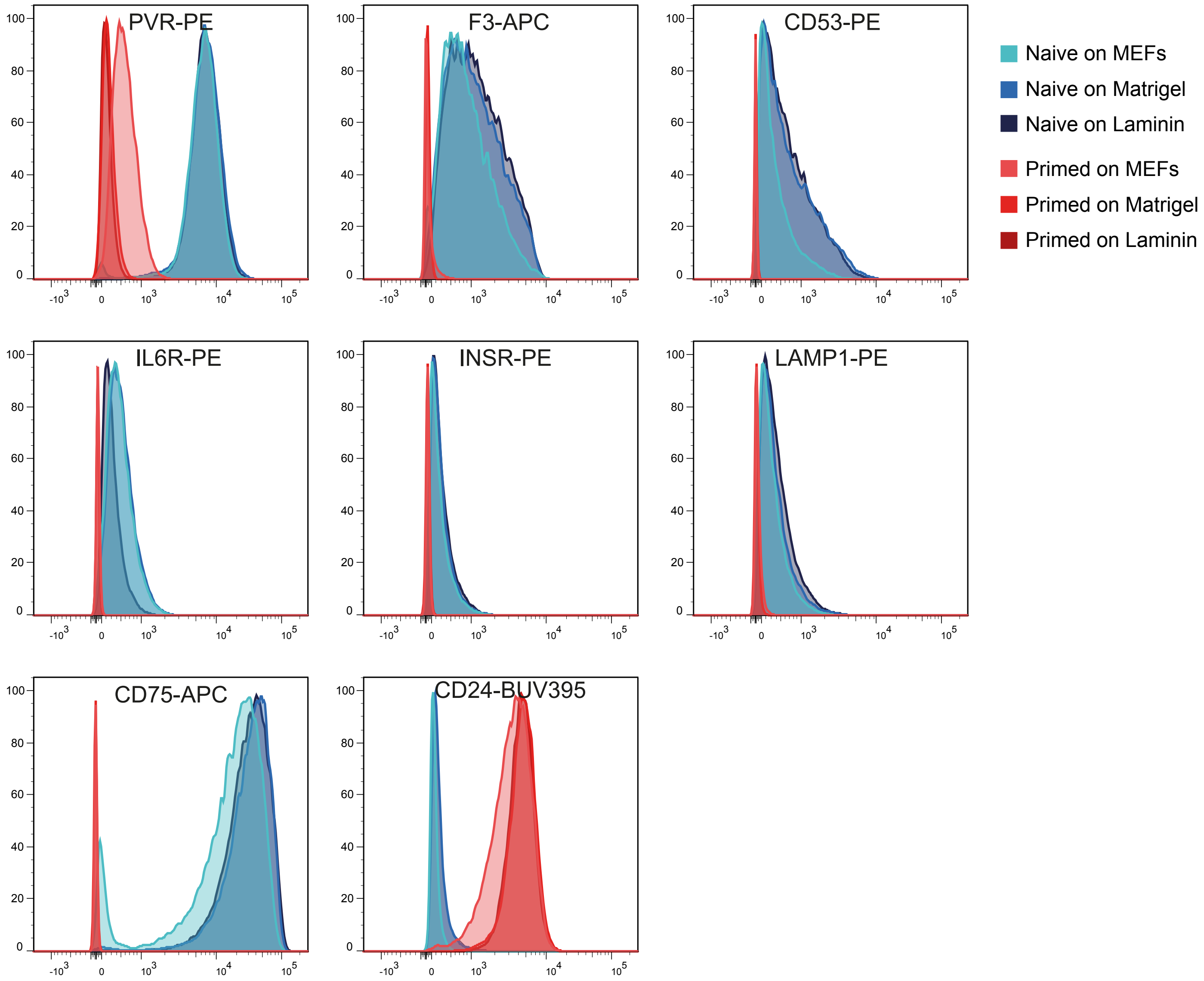

PVR-PE
F3-APC
CD53-PE
IL6R-PE
INSR-PE
LAMP1-PE
CD75-APC
CD24-BUV395
Naive on MEFs
Naive on Matrigel
Naive on Laminin
Primed on MEFs
Primed on Matrigel
Primed on Laminin

Figure S5. Transcriptional meta-analysis of cell-surface markers in several hPSC, human embryo and primate embryo datasets, Related to Figure 4.

Heat maps show the gene expression levels of identified cell-surface markers in multiple stem cell and developmental datasets. The “Human PSC” (Collier et al., 2017) data show transcript levels in naive and primed hPSC, and the “Human PSC capacitation” (Rostovskaya et al., 2019) data show transcript levels over a time course starting from naive hPSC at day 0. The “Human epiblast” (Xiang et al., 2020; Zhou et al., 2019) and “Primate epiblast” (Nakamura et al., 2016) datasets are derived from single-cell RNA-Seq libraries that have been filtered for epiblast cells and computationally merged so that all individual epiblast cells at the indicated developmental stage are included. ICM, inner cell mass; Pre, preimplantation epiblast; PostE, early postimplantation; PostL, late postimplantation.

Figure S5

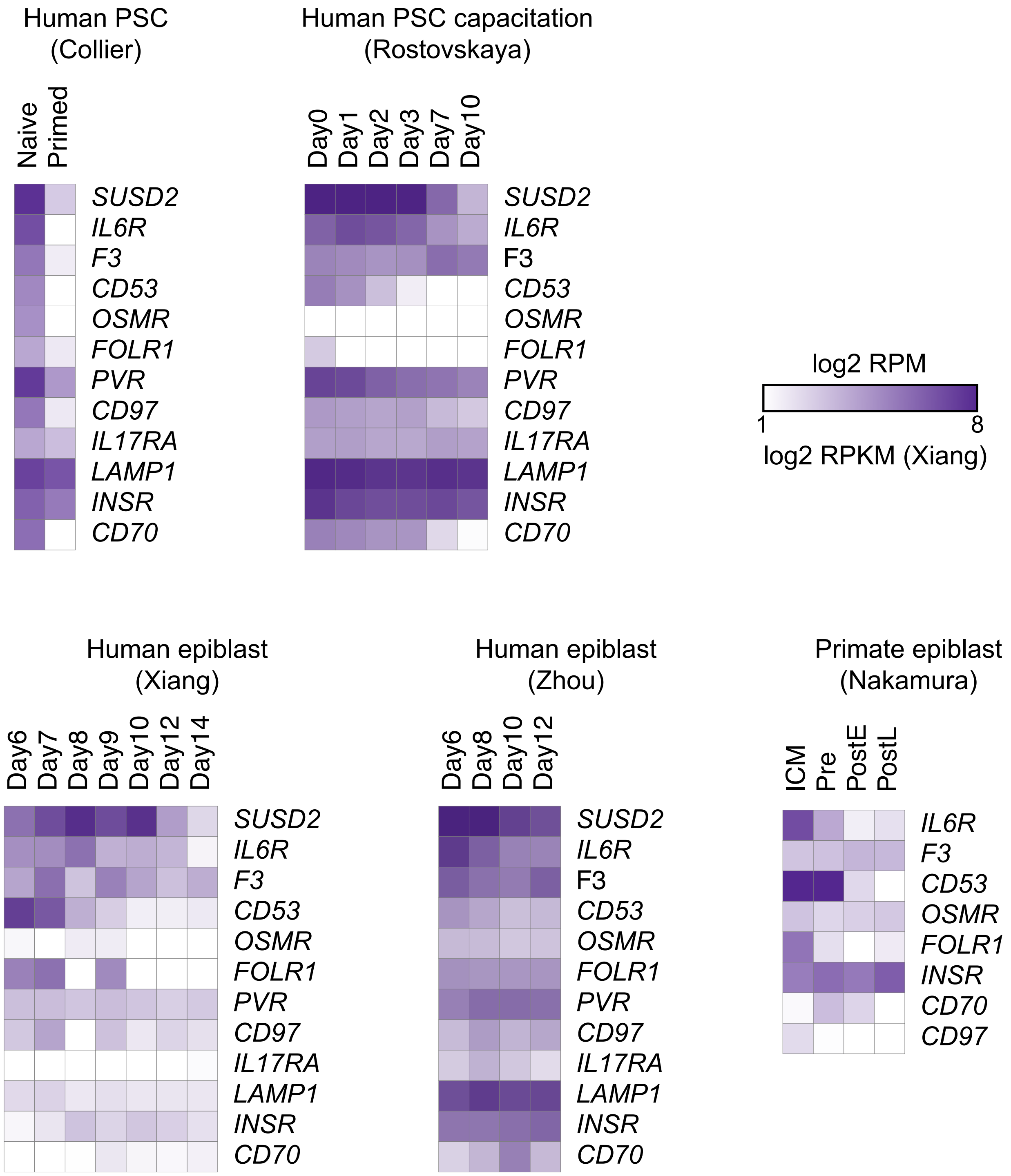

Human PSC
(Collier)
Naive
Primed
SUSD2
IL6R
F3
CD53
OSMR
FOLR1
PVR
CD97
IL17RA
LAMP1
INSR
CD70
Human PSC capacitation
(Rostovskaya)
Day0
Day1
Day2
Day3
Day7
Day10
SUSD2
IL6R
F3
CD53
OSMR
FOLR1
PVR
CD97
IL17RA
LAMP1
INSR
CD70
log2 RPM
1
8
log2 RPKM (Xiang)
Human epiblast
(Xiang)
Day6
Day7
Day8
Day9
Day10
Day12
Day14
SUSD2
IL6R
F3
CD53
OSMR
FOLR1
PVR
CD97
IL17RA
LAMP1
INSR
CD70
Human epiblast
(Zhou)
Day6
Day8
Day10
Day12
SUSD2
IL6R
F3
CD53
OSMR
FOLR1
PVR
CD97
IL17RA
LAMP1
INSR
CD70
Primate epiblast
(Nakamura)
ICM
Pre
PostE
PostL
IL6R
F3
CD53
OSMR
FOLR1
INSR
CD70
CD97

Figure S6. Characterisation of FOLR1 and SUSD2 expression in additional hPSC lines and conditions, Related to Figures 4, 5 and 6.

(A) Flow cytometry results showing that FOLR1 expression is higher in naive compared to primed hPSC in additional cell lines (left) and when maintained on different substrates (right). Results are representative of at least two biological replicates.

(B) RT-qPCR analysis of FOLR1, *FOLR2* and *FOLR3* gene expression levels in parental (WT) naive hPSC and FOLR1-deficient (KO) naive hPSC. Data show the mean ± SD of three biological replicates and were compared using an unpaired, two-sided t-test (ns, $q > 0.05$; **** $q < 0.0001$).

(C) Flow cytometry results showing that SUSD2 expression is higher in naive compared to primed hPSC in additional cell lines (left) and when maintained on different substrates (right). Results are representative of at least two biological replicates.

(D) Dot plots of flow cytometry analysis using SUSD2-PE conjugated antibody confirm the absence of SUSD2 signal in SUSD2-deficient (KO) and parental (WT) primed hPSC, and high SUSD2 signal in KO primed hPSC that express a SUSD2 rescue plasmid. Two different KO clones are shown (F5 and F10), each with two rescue clones. Wild-type naive hPSC provide a positive control for SUSD2 expression.

Figure S6

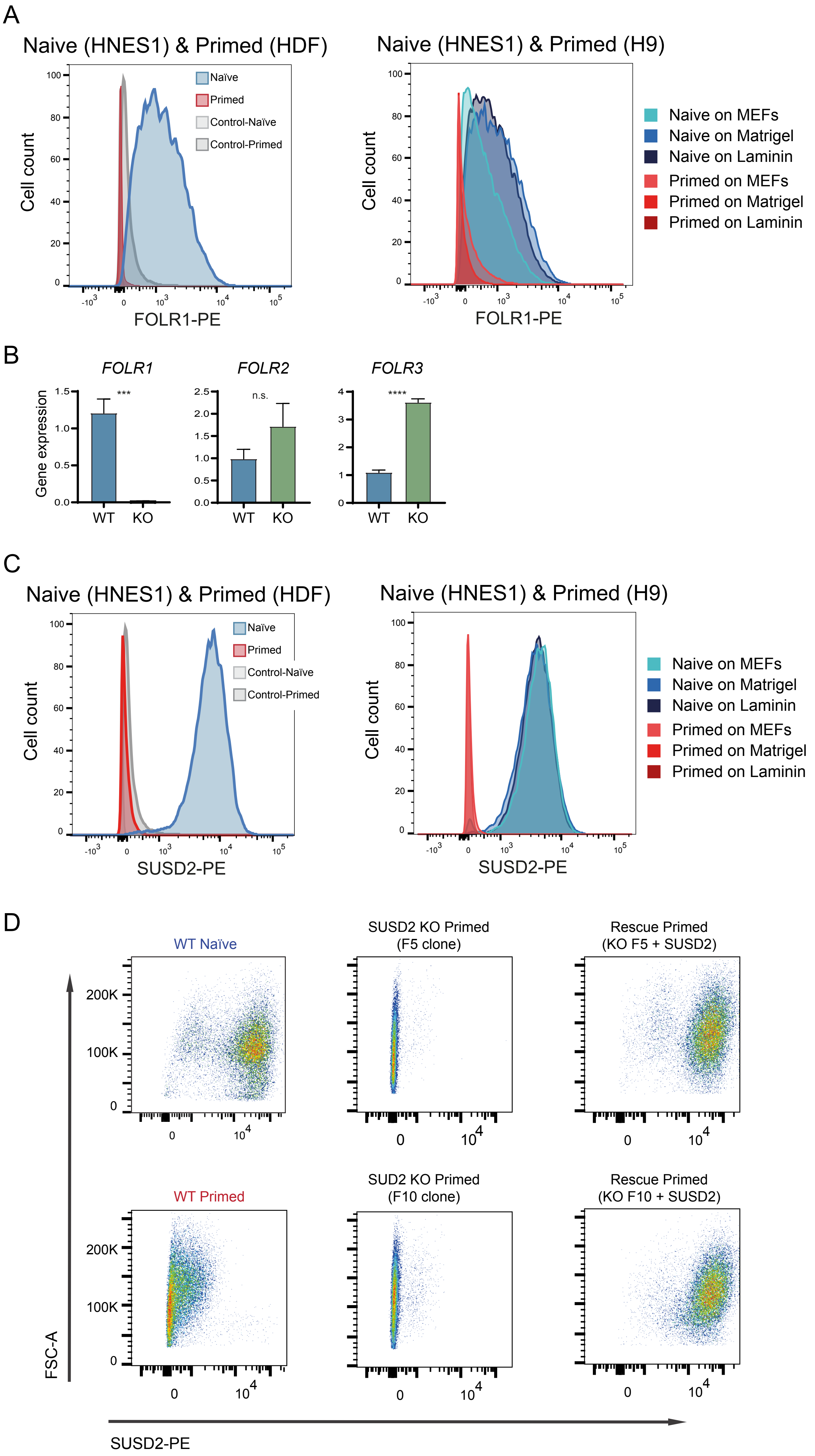

A
Naive (HNES1) & Primed (HDF)
Naïve
Primed
Control-Naïve
Control-Primed
Cell count
FOLR1-PE
Naive (HNES1) & Primed (H9)
Naive on MEFs
Naive on Matrigel
Naive on Laminin
Primed on MEFs
Primed on Matrigel
Primed on Laminin
B
FOLR1
FOLR2
FOLR3
Gene expression
***
n.s.
****
WT
KO
C
Naive (HNES1) & Primed (HDF)
SUSD2-PE
Naive (HNES1) & Primed (H9)
D
WT Naïve
SUSD2 KO Primed (F5 clone)
Rescue Primed (KO F5 + SUSD2)
WT Primed
SUD2 KO Primed (F10 clone)
Rescue Primed (KO F10 + SUSD2)
200K
100K
FSC-A
SUSD2-PE

SUPPLEMENTAL TABLES

Table S1.

Full dataset of cell-surface proteomes in naive and primed hPSC, Related to Figure 1.

Table S2.

List of GO terms that are associated with the detected cell-surface proteins, Related to Figure 2.

Table S3.

List of antibodies used, Related to Figure 4 and Supplemental Experimental Procedures.

SUPPLEMENTAL EXPERIMENTAL PROCEDURES

Cell culture

Naive H9 hPSC (kindly provided by Austin Smith, with permission from WiCell) were maintained in t2iLGö media as previously described (Takashima et al., 2014) in N2B27 (a 1:1 mixture of DMEM/F12 and Neurobasal, 0.5x N2 supplement, 0.5x B27 supplement, 2mM L-Glutamine, 1x Penicillin/Streptomycin (all from ThermoFisher Scientific), 0.1mM β-mercaptoethanol (Sigma-Aldrich)) supplemented with 1µM PD0325901, 1µM CHIR99021, 20ng/ml human LIF (all from WT-MRC Cambridge Stem Cell Institute) and 2µM Gö6983 (Tocris) on either a layer of irradiated mouse embryonic fibroblasts (MEF) seeded at a density of $2x10^6$ cells per 6-well plate or on Matrigel–coated plates (Corning).

HNES1 naive hPSC (Guo et al., 2016) (kindly provided by Austin Smith) were maintained in PXGL media as previously described (Guo et al., 2017) in N2B27 supplemented with 1µM PD0325901, 2µM XAV939 (Selleckchem), 2µM Gö6983 and 10 ng/mL human LIF. Cells were cultured on either a layer of irradiated MEF or on Matrigel-, Geltrex- (ThermoFisher Scientific) or Laminin-coated plates (iMatrix-511, Takara).

WIBR3 naïve hPSC cultured in 5iLA conditions were kindly provided by Rudolph Jaenisch and maintained as previously described (Theunissen et al., 2014, 2016) in N2B27 supplemented with 50µg/ml bovine serum albumin (ThermoFisher Scientific), 0.5% KnockOut Serum Replacement (KSR; ThermoFisher Scientific), 20ng/ml recombinant human LIF, 20ng/ml Activin A (WT-MRC Cambridge Stem Cell Institute), 1µM PD0325901, 1µM IM-12 (Sigma-Aldrich), 1µM WH-4-023 (A Chemtek), 0.5µM SB590885 (Sigma-Aldrich), 10µM Y-27632 (Cell Guidance Systems) on a MEF-layer seeded at a density of $2x10^6$ cells per 6-well plate.

All naïve hPSC were passaged by dissociation with Accutase (Thermo Fisher Scientific) every 3-4 days.

H9 and HDF primed hPSC were maintained on Vitronectin-coated plates (0.5µg/cm^2; ThermoFisher Scientific) in TeSR-E8 media (StemCell Technologies). Cells were passaged using 0.5mM EDTA (Sigma-Aldrich) every 4-5 days. For the initiation of reprogramming experiments, for culturing WIBR3 primed hPSCs and when making knockout cell lines, hPSC were maintained in primed hPSC media comprised of Advanced DMEM, 20% KSR, 2mM L-Glutamine, 1x Penicillin/Streptomycin, 0.1mM β-mercaptoethanol and 4ng/ml basic fibroblast growth factor (WT-MRC Cambridge Stem Cell Institute) on MEFs seeded at a density of $1x10^6$ cells per 6-well plate. Cells were passaged using 200U/ml Collagenase type IV (ThermoFisher Scientific).

For 5iLA reprogramming (Theunissen et al., 2014), primed hPSC cultured on MEF-coated plates were dissociated into single cells with Accutase and $2x10^5$ cells per 6-well were plated in KSR-containing primed hPSC media with 10µM Y-27632 onto MEF seeded at a density of $2x10^6$ cells per 6-well plate. The following day, media was changed to 5iLA and cells were passaged with Accutase on day 5 and on day 10. For reprogramming using Chemical Resetting (Guo et al., 2017), primed hPSC cultured on MEF-coated plates were dissociated into single cells with Accutase and $5x10^5$ cells per 10cm dish were plated in KSR-containing primed hPSC media supplemented with 10µM Y-27632. After two days, media was changed to N2B27 with 1µM PD0325901, 10ng/ml recombinant human LIF and 1mM valproic acid (Sigma-Aldrich). After an additional three days, media was changed to PXGL (N2B27 with 1µM PD0325901, 2µM XAV939, 2µM Gö6983 and 10ng/ml recombinant human LIF) and changed daily. Cells were passaged with Accutase on day 10 and thereafter maintained in tt2iLGö (N2B27 with 1µM PD0325901, 2µM XAV939, 2µM Gö6983, 10ng/ml human LIF and 0.3µM CHIR99021) until homogenous naive lines were obtained.

All hPSC were cultured in 5% O_2, 5% CO_2 at 37°C.

Targeted deletion of *FOLR1* and *SUSD2*

Dual gRNAs were designed using WGE (https://www.sanger.ac.uk/science/tools/wge) to excise an early exon that would cause a frameshift upon deletion. Sequences used for FOLR1 were TCTATTCGGGGCTGAGTTGCTGG and GGCAGACCTCAAGATAGTTCCGG, and for SUSD2 GCAGCCAGTCCAGTACCCGCCGG and TGGGGGCCCAGACCATCGAGAGG. Each gRNA sequence was incorporated into the U6 target gRNA expression vector (Mali et al., 2013) and synthesized as a gBlock by Integrated DNA Technologies. The gRNA gBlocks were sub-cloned into pCR2.1-TOPO (ThermoFisher Scientific) and verified by sequencing. Primed hPSC were dissociated into single cells using Accutase and $2x10^6$ cells were nucleofected with 5µg pCas9_GFP (Addgene plasmid #44719) and 2.5µg of each gRNA expression vector. After 48 hours, 10,000 GFP-positive single cells were isolated by flow cytometry and seeded onto MEF in a 10-cm tissue culture dish in primed hPSC media supplemented with 10µM Y-27632 for the first 24 hours. Individual clones were picked and expanded in 24-well plates, and genotyped using the primers SUSD2_For ATTCTAGCCCGTCAGTCAGG, SUSD2_Rev AGTGACACAGTGAAGGGGAT, FOLR1_For GGCATTTCATCCAGGACACC and FOLR1_Rev CTGCGCACTTGTTAAACCCT. Mutations were validated by DNA sequencing of TOPO cloned PCR products.

***SUSD2* rescue cell lines**

RNA was extracted using an RNeasy Mini Kit (QIAGEN) and reverse transcribed using SuperScript II Reverse Transcriptase (ThermoFisher Scientific). The full-length *SUSD2* coding sequence was amplified from the cDNA using HotStarTaq DNA polymerase (QIAGEN) using the primer sequences ACGACGCGTGCCACCATGAAGCCAGCCCTCCTG and ATAAGAATGCGGCCGCTCAGGGCTGTGCACCC. A 2.1Kb PCR product was excised and cloned into a pCAG expression plasmid (Niwa et al., 1991) using *XhoI* and *NotI* enzymes (ThermoFisher Scientific). The resultant plasmid 'pCAG-SUSD2-ires-puro' was confirmed by DNA sequencing. The plasmid was transfected into two *SUSD2*-deficient primed hPSC lines using

GeneJuice (MerckMillipore). Cells were treated with 1µg/ml puromycin for 48h and resistant colonies were expanded. The expression of SUSD2 in the rescue cells was confirmed by flow cytometry.

Cell proliferation assay

Naïve hPSC were dissociated with Accutase and seeded at 10,000 cells per well on 12-well plates coated with Matrigel. Four wells were seeded per condition and cells were maintained in t2iLGö. After 24h, the JAK inhibitor (CalBiochem #420099) was added at 1µM in t2iLGö for a 5 day treatment and media was refreshed daily. JAK inhibitor-treated cells and controls were harvested with Accutase every 24h, mixed with trypan blue and viable cells were counted using a hemocytometer.

Plasma membrane profiling (PMP)

Cell labelling

PMP was performed as described previously (Weekes et al., 2012). Briefly, $1x10^7$ cells per condition (n=3) were harvested with Accutase or 0.5mM EDTA and washed with PBS. Cell pellets were resuspended in biotinylation buffer (97µM aminooxy-biotin, 1mM sodium periodate, 0.093% aniline in PBS pH 6.7) and incubated for 30min at 4°C in the dark. This step labels cell-surface glycans by introducing an aldehyde at the C-7 position of exposed sialic acids followed by ligation with aminooxy-biotin (Zeng et al., 2009). Labelling was quenched with 1mM glycerol, cells were washed with PBS pH 7.4 and stored at -80°C until further use.

Enrichment of cell-surface proteins

Cell pellets were thawed on ice, resuspended in lysis buffer (10mM Tris-HCl, 150mM NaCl, 1% Triton X-100 with cOmplete protease inhibitor cocktail from Roche/Sigma) and incubated for 30min at 4°C with rotation. Lysates were cleared by two rounds of centrifugation at 13,000g for 10min, and supernatants

were transferred to fresh LoBind tubes and mixed with pre-equilibrated streptavidin beads (~3h at 4°C with rotation). After discarding the supernatant, beads were washed with 8ml of lysis buffer followed by 4ml wash with 0.5% SDS in 50mM Triethylammonium bicarbonate (TEAB). Proteins bound to the beads were reduced with 10mM tris(2-carboxyethyl)phosphine (TCEP) and alkylated with 20mM iodoacetamide (IAM) for 30min at room temperature (RT) with shaking in the dark. Beads were washed with further 4ml of SDS buffer, 8ml of urea buffer (6M urea in 50 mM TEAB, pH 8.5), 2ml of 50mM TEAB and transferred to Eppendorf tubes for overnight trypsin digestion at 37°C with shaking.

TMT labelling

Samples were centrifuged to pellet the beads and supernatants containing the peptides were transferred to fresh tubes and lyophilised in a vacuum centrifuge. Samples were resuspended in 50µl 50mM TEAB before addition of TMT tags (20µl in acetonitrile, ACN). Samples were labelled for 1h at RT with shaking and excess TMT was quenched with 0.3% hydroxylamine for a further 20min. TMT-labelled samples were then pooled together and lyophilised as before.

High pH RP fractionation

Lyophilised samples were resuspended in 16µl 0.2% NH_3 for high pH reverse phase fractionation on UltiMate 3000 system (ThermoFisher Scientific). In-house made microcolumn (0.53 x 200mm packed with ReproSil-Pur C18-AQ resin, 3µm particle size (dr. Maisch) was used for peptide separation at 20µl/min using linear gradient 0-40% ACN over 30min. Eluted peptides were manually collected into 1min fractions and stored at -80°C until further use.

nLC MS acquisition

Peptide-enriched fractions were analysed in nLC MSMS mode using UltiMate 3000 liquid chromatography system (ThermoFisher Scientific) interfaced via EASY-Spray™ Source onto Orbitrap Fusion™ Lumos™ Tribrid™ mass spectrometer (ThermoFisher Scientific). 50% of each fraction was used and samples were loaded onto pre-column (Acclaim PepMap100, 100µm inner diameter, 2cm long, packed with 5µm C18 particles) in 0.1% TFA at a flow rate of 8µl/min. Peptides were eluted from the precolumn and separated over analytical column (EASY-Spray, PepMap RSLC 75µm ID, 50cm long, packed with 2µm C18 particles) using a linear gradient of solvent B (80% ACN, 0.1% FA), from 2 to 45% B at 300nl/min over 90min at 40°C. Eluted peptides were ionised using 2.1kV voltage and introduced to mass spectrometer as gas-phase ions. MS1 scans were triggered when threshold of 4E5 ions or 50 ms was reached and were acquired in Orbitrap with 120000 resolution at 375-1800 m/z range. The most intense ions from each MS1 spectrum were isolated in the quadrupole using 0.7 m/z window and fragmented using CID with 35% collision energy. MS2 scans of fragment ions were triggered when threshold of 1E4 ions or 150ms (75ms for more abundant fractions) was reached and were acquired in Ion Trap starting at 120 m/z. Fragmented precursors were excluded from repeated analysis for max 30s (per 120min gradient). Top 10 fragment ions were isolated in Ion Trap using 2 m/z isolation window and fragmented using HCD with 65% collision energy. MS3 scans were triggered when threshold of 1E5 ions or 105ms was reached and were acquired in Orbitrap with 50000 resolution and scan range 100-500 m/z. MS1-3 spectra were recorded in Xcalibur 3.0.

Data processing

Data were processed in Proteome Discoverer 2.1 (ThermoFisher Scientific). Raw data from all the fractions were combined and searched against human reference proteome (71579 entries, October 2017) and GPM database of common contaminants (57 entries) using Mascot server (Matrix Science) as well as the reverse decoy database and use of Percolator for refinement and FDR calculation (Käll et al., 2007). Trypsin was

used as protease with a maximum of two missed cleavage sites allowed. Peptide mass tolerance was set to 10ppm and fragment mass tolerance was 20mmu. Carbamidomethylation of cysteines and TMT modification of peptide N-terminus and lysines were used as fixed modifications and oxidation of methionines was used as variable modification. Only high confidence peptides were used (0.01 target peptide FDR) and minimum of one unique peptide was considered for successful protein identification. Reporter ion intensities were extracted from MS3 spectra using average reporter signal to noise threshold of 10 and isolation interference lower than 50%. We excluded the third replicates of EDTA and Accutase primed hPSC samples from all downstream analyses due to poor sample quality leading to a low correlation with other samples. The final dataset used, therefore, contains three naïve hPSC samples and four primed hPSC samples.

Significance analysis

Only unique peptides were used to calculate protein abundances using RRollup function within InfernoRDN v1.1.6452.22004 (Polpitiya et al., 2008). The final list of protein abundances was used for statistical analysis with an in-house R script for LIMMA and Benjamini-Hochberg correction for multiple hypothesis testing.

Analysis of transcript and protein abundance

To express protein abundances in individual samples, we combined the label-free quantitative method Top3 (Grossmann et al., 2010; Silva et al., 2006) and TMT reporter ion intensities. Top3 protein abundance was multiplied by TMT fractions (individual TMT intensity divided by the sum) to calculate protein abundances of individual samples. Transcriptional datasets were downloaded from GEO. Heatmaps were made using Morpheus (https://software.broadinstitute.org/morpheus).

Gene ontology enrichment analysis

Gene ontology analysis was performed in EnrichR (Chen et al., 2013; Kuleshov et al., 2016) using 1154 significantly different proteins against a background list of total human proteins using default settings. For GO Biological Process, the top 200 enriched terms (ranked by the descending combined score) were uploaded to ReviGo (Supek et al., 2011) for grouping and the final plot was made in R.

Quantitative mass spectrometry analysis of DNA methylation

Relative abundances of mC and hmC in genomic DNA samples were measured and analysed as described previously (Takashima et al., 2014).

Flow cytometry

Conjugated antibodies (Table S3) were mixed with 50µL Brilliant stain buffer (BD Biosciences) and applied to 50µL of single cells (1-5x10^5 cells per reaction). Cells were incubated for 30min at 4°C in the dark, washed with flow buffer (2% FBS in PBS) and centrifuged at 300xg for 3min. Where applicable, MEF in the samples were identified by reactivity to CD90.2-APC-Cy7 antibody. Cells were resuspended in buffer with DAPI or 7-AAD (BD Biosciences) and analysed with a BD LSRFortessa cell analyzer (BD Biosciences). Data was analysed using FlowJo v10.5.3 software (FlowJo, LLC).

Immunofluorescence microscopy

Cells were fixed with 2% paraformaldehyde for 10min, washed three times with PBS, and incubated with either FOLR1-PE (1:20) or SUSD2-PE (1:100) antibodies (Table S3) overnight at 4°C in the dark. Cells were washed with PBS, labelled with DAPI and imaged on a Nikon Ti-E.

Histone protein extraction

Cell pellets were thawed on ice, washed with ice-cold PBS, resuspended in 1ml ice-cold 0.2M H_2SO_4 and incubated on ice for 30min. Samples were centrifuged at 14,000g for 2min at 4°C and the supernatant was transferred to a new LoBind tube. TCA (333µl) was added and the samples were incubated on ice for 30min. Precipitated proteins were collected by centrifugation at 14,000g for 10min at 4°C. Protein pellets were washed twice with acetone and resuspended in 100mM Tris-HCl pH 8 supplemented with protease inhibitors. Histone extracts were cleared by centrifugation as before and stored at -80°C.

Western Blot analysis

Cell pellets were resuspended in ice-cold RIPA buffer (50mM Tris-HCl, pH 8, 150 mM NaCl, 1% NP-40, 0.5% SDC, 0.1% SDS supplemented with protease and PhosSTOP phosphatase inhibitor tablets from Roche/Sigma) and incubated for 30min at 4°C with rotation. Lysates were cleared by centrifugation for 15min at 14,000g at 4°C and supernatants were transferred to LoBind tubes. An aliquot was used to determine protein concentration using the Pierce BCA assay (ThermoFisher Scientific). For SDS-PAGE Bolt system was used, with 10% Bolt Bis-Tris Plus gels. Protein lysates (30µg) were prepared in Bolt LDS sample buffer with 50mM DTT and incubated for 10min at 70°C before loading into the gel. Electrophoresis was in MES running buffer for 30min at 200V. Gel was transferred onto PVDF membrane using XCell SureLock™ Mini-Cell blot module following manufacturer’s instructions. Membrane blocking was 1h at RT in 5% milk in TBST (or 5% BSA for pSTAT3). Antibody solutions were prepared in blocking buffer, incubation with primary antibodies (Table S3) was overnight at 4°C and secondary antibodies for 1h at RT. Signal was detected using Clarity Western ECL substrate (BioRad), except for the histone blots which were probed with fluorophore-conjugated secondary antibodies and imaged using Li-COR system.

Quantitative RT-PCR

Cells were lysed in RLT buffer, RNA was extracted using an RNeasy Mini Kit (QIAGEN) and reverse transcribed using Quantiscript Reverse Transcriptase (QIAGEN). cDNA (250ng) was analysed using SYBR Green Master Mix (Sigma). Primer sequences are available on request.

SUPPLEMENTAL REFERENCES

Chen, E.Y., Tan, C.M., Kou, Y., Duan, Q., Wang, Z., Meirelles, G.V., Clark, N.R., and Ma'ayan, A. (2013). Enrichr: interactive and collaborative HTML5 gene list enrichment analysis tool. BMC Bioinformatics *14*, 128.

Collier, A.J., Panula, S.P., Schell, J.P., Chovanec, P., Plaza Reyes, A., Petropoulos, S., Corcoran, A.E., Walker, R., Douagi, I., Lanner, F., et al. (2017). Comprehensive Cell Surface Protein Profiling Identifies Specific Markers of Human Naive and Primed Pluripotent States. Cell Stem Cell *20*, 874–890.e7.

Di Stefano, B., Ueda, M., Sabri, S., Brumbaugh, J., Huebner, A.J., Sahakyan, A., Clement, K., Clowers, K.J., Erickson, A.R., Shioda, K., et al. (2018). Reduced MEK inhibition preserves genomic stability in naive human embryonic stem cells. Nat. Methods *15*, 732–740.

Grossmann, J., Roschitzki, B., Panse, C., Fortes, C., Barkow-Oesterreicher, S., Rutishauser, D., and Schlapbach, R. (2010). Implementation and evaluation of relative and absolute quantification in shotgun proteomics with label-free methods. J. Proteomics *73*, 1740–1746.

Guo, G., von Meyenn, F., Santos, F., Chen, Y., Reik, W., Bertone, P., Smith, A., and Nichols, J. (2016). Naive Pluripotent Stem Cells Derived Directly from Isolated Cells of the Human Inner Cell Mass. Stem Cell Reports *6*, 437–446.

Guo, G., von Meyenn, F., Rostovskaya, M., Clarke, J., Dietmann, S., Baker, D., Sahakyan, A., Myers, S., Bertone, P., Reik, W., et al. (2017). Epigenetic resetting of human pluripotency. Development *144*, 2748–2763.

Käll, L., Canterbury, J.D., Weston, J., Noble, W.S., and MacCoss, M.J. (2007). Semi-supervised learning for peptide identification from shotgun proteomics datasets. Nat. Methods *4*, 923–925.

Kuleshov, M.V., Jones, M.R., Rouillard, A.D., Fernandez, N.F., Duan, Q., Wang, Z., Koplev, S., Jenkins, S.L., Jagodnik, K.M., Lachmann, A., et al. (2016). Enrichr: a comprehensive gene set enrichment analysis web server 2016 update. Nucleic Acids Res. *44*, W90–W97.

Mali, P., Yang, L., Esvelt, K.M., Aach, J., Guell, M., DiCarlo, J.E., Norville, J.E., and Church, G.M. (2013). RNA-guided human genome engineering via Cas9. Science *339*, 823–826.

Nakamura, T., Okamoto, I., Sasaki, K., Yabuta, Y., Iwatani, C., Tsuchiya, H., Seita, Y., Nakamura, S., Yamamoto, T., and Saitou, M. (2016). A developmental coordinate of pluripotency among mice, monkeys and humans. Nature *537*, 57–62.

Niwa, H., Yamamura, K., and Miyazaki, J. (1991). Efficient selection for high-expression transfectants with a novel eukaryotic vector. Gene *108*, 193–199.

Polpitiya, A.D., Qian, W.-J., Jaitly, N., Petyuk, V.A., Adkins, J.N., Camp, D.G., 2nd, Anderson, G.A., and Smith, R.D. (2008). DAnTE: a statistical tool for quantitative analysis of -omics data. Bioinformatics *24*, 1556–1558.

Rostovskaya, M., Stirparo, G.G., and Smith, A. (2019). Capacitation of human naïve pluripotent stem cells for multi-lineage differentiation. Development *146*.

Silva, J.C., Gorenstein, M.V., Li, G.-Z., Vissers, J.P.C., and Geromanos, S.J. (2006). Absolute quantification of proteins by LCMSE: a virtue of parallel MS acquisition. Mol. Cell. Proteomics *5*, 144–156.

Supek, F., Bošnjak, M., Škunca, N., and Šmuc, T. (2011). REVIGO summarizes and visualizes long lists of gene ontology terms. PLoS One *6*, e21800.

Theunissen, T.W., Powell, B.E., Wang, H., Mitalipova, M., Faddah, D.A., Reddy, J., Fan, Z.P., Maetzel, D., Ganz, K., Shi, L., et al. (2014). Systematic Identification of Culture Conditions for Induction and Maintenance of Naive Human Pluripotency. Cell Stem Cell *15*, 524–526.

Theunissen, T.W., Friedli, M., He, Y., Planet, E., O'Neil, R.C., Markoulaki, S., Pontis, J., Wang, H., Iouranova, A., Imbeault, M., et al. (2016). Molecular Criteria for Defining the Naive Human Pluripotent State. Cell Stem Cell *19*, 502–515.

Weekes, M.P., Antrobus, R., Talbot, S., Hör, S., Simecek, N., Smith, D.L., Bloor, S., Randow, F., and Lehner, P.J. (2012). Proteomic Plasma Membrane Profiling Reveals an Essential Role for gp96 in the Cell Surface Expression of LDLR Family Members, Including the LDL Receptor and LRP6. Journal of Proteome Research *11*, 1475–1484.

Xiang, L., Yin, Y., Zheng, Y., Ma, Y., Li, Y., Zhao, Z., Guo, J., Ai, Z., Niu, Y., Duan, K., et al. (2020). A developmental landscape of 3D-cultured human pre-gastrulation embryos. Nature *577*, 537–542.

Zeng, Y., Ramya, T.N.C., Dirksen, A., Dawson, P.E., and Paulson, J.C. (2009). High-efficiency labeling of sialylated glycoproteins on living cells. Nat. Methods *6*, 207–209.

Zhou, F., Wang, R., Yuan, P., Ren, Y., Mao, Y., Li, R., Lian, Y., Li, J., Wen, L., Yan, L., et al. (2019). Reconstituting the transcriptome and DNA methylome landscapes of human implantation. Nature *572*, 660–664.

Takashima, Y., Guo, G., Loos, R., Nichols, J., Ficz, G., Krueger, F., Oxley, D., Santos, F., Clarke, J., Mansfield, W., et al. (2014). Resetting Transcription Factor Control Circuitry toward Ground-State Pluripotency in Human. Cell *158*, 1254–1269.
